# Bell-Evans model and steered molecular dynamics in uncovering the dissociation kinetics of ligands targeting G-protein-coupled receptors

**DOI:** 10.1038/s41598-022-20065-2

**Published:** 2022-09-24

**Authors:** Muhammad Jan Akhunzada, Hyun Jung Yoon, Indrajit Deb, Abdennour Braka, Sangwook Wu

**Affiliations:** 1 R&D Center, PharmCADD Co. Ltd., 12F, 331, Jungang-daero, Dong-gu, Busan, 48792 Republic of Korea; 2 Department of Physics, Pukyong National University, Busan, 48513 Republic of Korea

**Keywords:** Biophysics, Computational biology and bioinformatics, Drug discovery

## Abstract

Recently, academic and industrial scientific communities involved in kinetics-based drug development have become immensely interested in predicting the drug target residence time. Screening drug candidates in terms of their computationally predicted residence times, which is a measure of drug efficacy in vivo, and simultaneously assessing computational binding affinities are becoming inevitable. Non-equilibrium molecular simulation approaches are proven to be useful in this purpose. Here, we have implemented an optimized approach of combining the data derived from steered molecular dynamics simulations and the Bell-Evans model to predict the absolute residence times of the antagonist ZMA241385 and agonist NECA that target the A2A adenosine receptor of the G-protein-coupled receptor (GPCR) protein family. We have predicted the absolute ligand residence times on the timescale of seconds. However, our predictions were many folds shorter than those determined experimentally. Additionally, we calculated the thermodynamics of ligand binding in terms of ligand binding energies and the per-residue contribution of the receptor. Subsequently, binding pocket hotspot residues that would be important for further computational mutagenesis studies were identified. In the experiment, similar sets of residues were found to be in significant contact with both ligands under study. Our results build a strong foundation for further improvement of our approach by rationalizing the kinetics of ligand unbinding with the thermodynamics of ligand binding.

## Introduction

Conventional lead optimization methods in drug development through high-throughput screening rely on biochemical and biophysical assays of drug-target binding affinity under in vitro thermodynamic equilibrium conditions^1,2^. Notwithstanding their importance in determining the potency of a drug candidate, the traditional equilibrium binding affinity parameters, such as the dissociation constant (*K*_*d*_), inhibition constant (*K*_*i*_), half-maximal inhibitory concentration (IC_50_), and effector concentration (EC_50_), are arguable in terms of their effectiveness in vivo^3^. The equilibrium dissociation constant (*K*_*d*_) of a drug-target complex is a major determinant of the concentration or dose of a drug required for sufficient drug-target interaction. A lower *K*_*d*_ corresponds to an increased amount of the drug, which in turn suggests a better medicinal effect of the drug candidate but may trigger off-target interactions. In a drug-target binding equilibrium (Eq. 1), the ratio of the association rate constant (*k*_*on*_) to the dissociation rate constant (*k*_*off*_) describes the binding constant (*K*_*b*_), the reciprocal of which defines the dissociation constant (*K*_*d*_), as shown in Eq. (2).

1 $$\begin{gathered} Drug + Traget\mathop {\mathop \rightleftharpoons \limits^{{k_{on} }} }\limits_{{k_{off} }} Drug \cdot Target \hfill \\ K_{b} = { }\frac{{k_{on} }}{{k_{off} }} = { }\frac{{\left[ {Drug \cdot Target} \right]}}{{\left[ {Drug\left] \cdot \right[Target} \right]}} \hfill \\ \end{gathered}$$

In Eq. (1), *k*_*on*_, *k*_*off*_ and *K*_*b*_ are the association rate constant, dissociation rate constant and binding constant, respectively.

2 $$K_{d} = { }\frac{1}{{K_{b} }} = { }\frac{{k_{off} }}{{k_{on} }}$$

Equation (2) indicates that *K*_*d*_ would be directly correlated with *k*_*off*_, considering that chemically similar drug candidates will possess similar *k*_*on*_^4–6^. In principle, slower unbinding or lower *k*_*off*_ would correspond to better drug efficacy, ensuring long-lasting pharmacological activity. Intriguingly, during the last decade, the pharmaceutical community has become interested in measuring the mean lifetime or the mean residence time (RT) of a drug-target complex. The RT can be defined as the time over which the ligand stays bound in the binding pocket of a receptor and can be expressed as the reciprocal of the dissociation rate constant (*k*_*off*_). Both the RT and *k*_*off*_ have already been proven to be more reliable metrics for determining the pharmacological activity of drug candidates in terms of the therapeutic index, dosing, toxicity, and selectivity of drug molecules under open, non-equilibrium *in vivo* conditions compared to the drug-target binding affinity^3^. *K*_*d*_ determines the pharmacological activity of a drug candidate, whereas RT determines the lifetime of the activity. Increased longevity of the pharmacological activity is of great interest in order to keep the pathological activity of the receptor suspended for a longer period of time. Therefore, screening drug candidates in terms of their RT would be a promising approach in kinetics-driven drug design campaigns, suggesting that the binding affinities and RTs should be simultaneously assessed for the selection of efficient drug candidates. Estimation of the RT has now become a routine in vitro measurement using surface plasmon resonance spectroscopy, atomic force spectroscopy, stopped-flow circular dichroism spectroscopy, kinetic capillary electrophoresis, and radiolabeling combined with filtration or dialysis^7^. The RT helps compare the pharmacodynamic-pharmacokinetic durability of ligand hits during the lead optimization stage of drug development.

An in-depth understanding of the drug-target interaction equilibrium requires a complete characterization of the thermodynamics and kinetics of the interactions. Extensive efforts have already been devoted to developing efficient computational methods for capturing the thermodynamics of drug-target interactions in terms of binding energy calculations using molecular docking and molecular dynamics simulation approaches. Molecular docking approaches, despite their simplicity and speed, have shortcomings considering the effect of the explicit solvent environment and configurational entropy of the systems^8^. The widely explored molecular dynamics (MD) simulation approaches, such as the Poisson–Boltzmann or generalized Born surface area (MMPBSA/MMGBSA) approaches, and alchemical methods, such as thermodynamic integration (TI) and free energy perturbation (FEP), demand sufficiently longer simulations to be carried out and complicated postprocessing to estimate the ligand binding energies^8–11^. Umbrella sampling, on the other hand, suffers from the challenges associated with a priori knowledge of reaction coordinates^8^. Regarding the prediction of drug unbinding kinetic constants in accelerating drug candidate selection, computational approaches such as the application of Markov state models to unbiased MD simulations, smoothed potential MD simulations, and enhanced sampling algorithms such as weighted ensemble methods, milestoning, transition path sampling, steered MD, scaled and selectively scaled MD, random acceleration MD, adiabatic-bias MD simulations, and conformational flooding have become very promising^12^.

G-protein-coupled receptors (GPCRs), some of the important and leading therapeutic targets^13–19^, are the largest family of integral membrane proteins and are generally characterized by the presence of seven transmembrane alpha helices separated by alternating extracellular and intracellular loops. Despite multibillion drug sales in the pharmaceutical industry, GPCRs are some of the most underexploited drug targets, as only 10% of GPCRs make it to the drug industry^15,16^. One of the main reasons behind this is the lack of understanding of the atomistic details of the GPCR-ligand interactions in terms of their binding kinetics and thermodynamics, which are the definitive parameters in the clinical success of any drug-target system. Studies have already reported that small-molecule drugs targeting GPCRs and related protein receptors show a high correlation between their efficacy and the RT^20–23^. Adenosine receptors (ARs) are generally classified into four subtypes, A1, A2A, A2B, and A3, and are activated by extracellular adenosine. These receptors are potential therapeutic targets for various conditions, such as sleep disorders, cancer, and dementia, due to their involvement in physiological processes, such as sleep regulation, angiogenesis, and modulation of the immune system^24^. The A2A AR is one of the best structurally characterized GPCRs, with more than 30 crystal structures available^25^.

**Figure 1:**
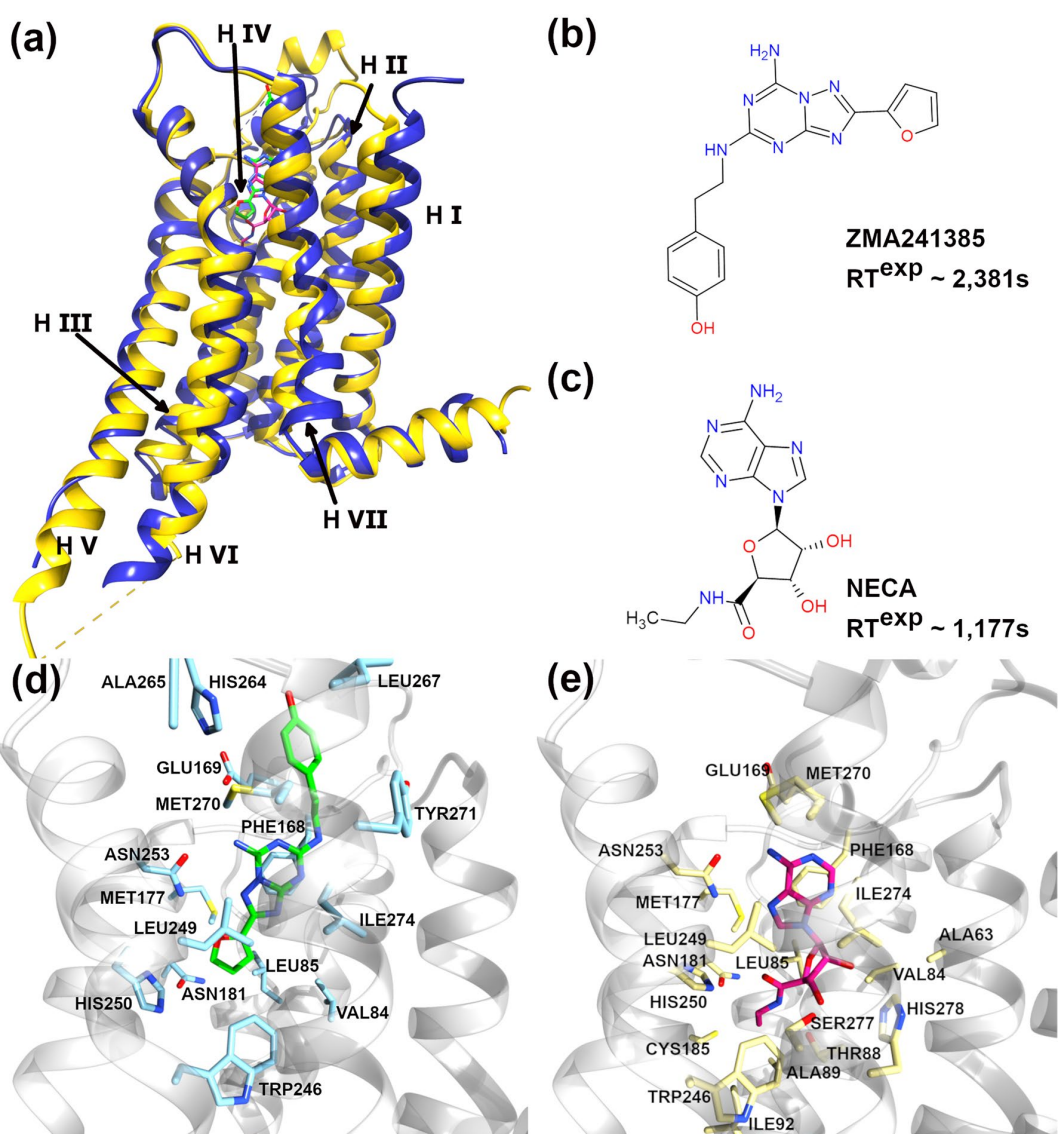
(**a**) The A2A AR-ligand complexes embedded into the 1-palmitoyl-2oleoyl-sn-glycero-3-phosphocholine (POPC) lipid bilayer; the A2A ARs corresponding to the PDB ids 3EML and 2YDV are shown in blue and yellow, respectively. The receptor helix (H) numbers are indicated in roman numbers. Experimentally determined RTs (RT^exp^) of ligands (**b**) ZMA241385 (4-(2-[7-amino-2-(2-furyl)-[1,2,4]triazolo[2,3-a][1,3,5]triazin-5-ylamino]ethyl)-phenol) and (**c**) NECA (N-Ethyl-5’-Carboxyamido Adenosine) are indicated. The binding pocket residues of the A2A AR within 5 Å of the ligands (**d**) ZMA241385 (green) and (**e**) NECA (magenta) are shown in blue and yellow sticks, respectively.

In this study, we develop an efficient computational approach for the prediction of the absolute RTs of drug candidates that target the A2A AR of the GPCR protein family (Fig. 1). Here, we implemented and tested our strategy of using steered molecular dynamics (SMD) simulations and the Bell-Evans model^26–30^, which relates the unbinding force (*F*_*R*_) to the parameters associated with the kinetics and energetics of ligand–receptor systems, as shown in Eq. (3).

3 $$F_{R} = \frac{{k_{B} T}}{{x_{b} }}ln\frac{{F^{\prime}x_{b} }}{{k_{b} Tk_{off} }}$$

**Figure 2:**
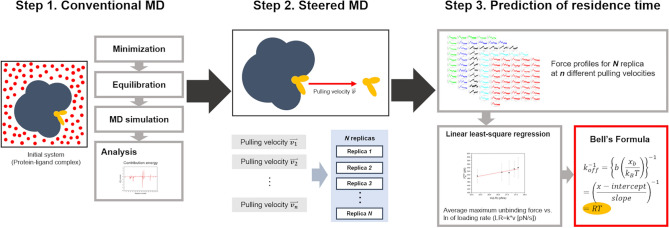
Workflow used in this work for the estimation of energetics and residence time (RT). In step 1, conventional molecular dynamics (MD) simulation was performed on the initial solvated system; subsequently energy calculations were performed on the simulated trajectory. In step 2, the final coordinate from the conventional MD simulation was subjected to steered molecular dynamics (SMD) simulations with different pulling velocities (*v*) and at a fixed spring force constant (*k*). At each of the pulling velocities SMD simulations were carried out in multiple replicas by reassigning the velocities on the starting structure. In step 3, post-processing of the SMD simulation data was carried out by obtaining the unbinding force profiles along the SMD simulation trajectories and employing the Bell-Evans model.

In Eq. (3), *k*_*B*_ is Boltzmann’s constant, *T* is the absolute temperature, *x*_*b*_ is the reaction coordinate corresponding to the separation between the bound and the transition state, projected along the direction of the applied force, *F*^′^ is the loading rate (LR), defined as the product of the stiffness of the force transducer (*k*) and velocity (*v*), and *k*_*off*_ is the dissociation rate constant at equilibrium. We predicted the dissociation rate constant (*k*_*off*_) values and the corresponding RT values of the ZMA241385 and NECA ligands that bind to the A2A AR of the GPCR protein family. In addition to predicting the kinetic parameters, the thermodynamics of GPCR-ligand interactions were extensively explored in our study. The MMPBSA^31^ approach-based binding energies of the ligands to the A2A AR and the interaction energies of these ligands with the binding pocket residues were estimated. Based on these energetics, structural hotspots in the A2A AR were identified. The results from this work build a strong foundation for further improvement of our approach of predicting the ligand dissociation rate constant (*k*_*off*_) by rationalizing the kinetics of ligand unbinding with the thermodynamics of ligand binding. The steps involved in our approach for predicting the absolute RT and energetics are depicted in Fig. 2.

## Results

### Prediction of the absolute residence time (RT) from unbinding steered molecular dynamics (SMD) simulations

**Figure 3:**
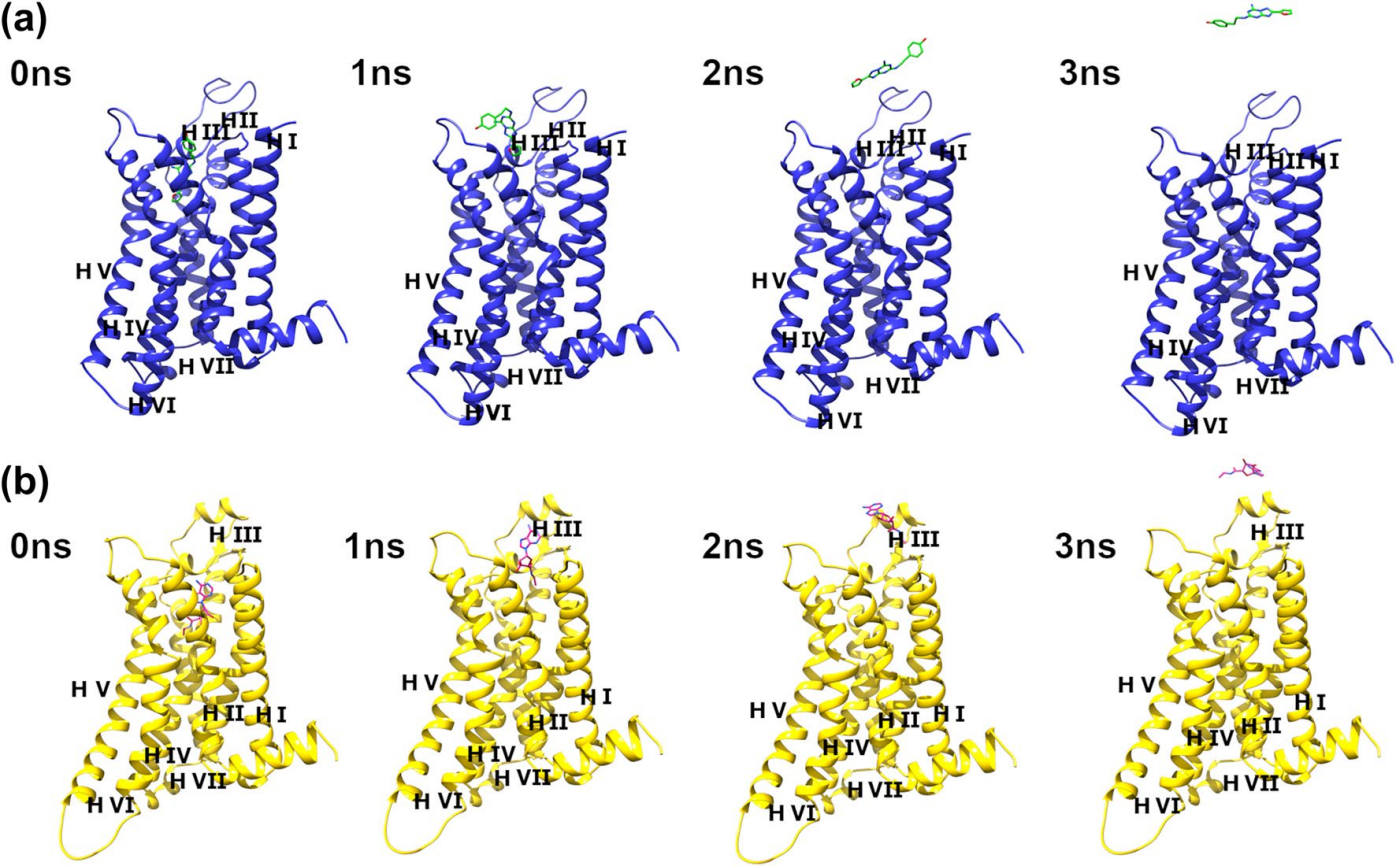
Snapshots at different time intervals from a representative SMD trajectory showing the unbinding of ligands with a pulling velocity of 0.0010 nm/ps. (**a**) Global view of the A2A AR (blue) in complex with the ligand ZMA241385 (green). (**b**) Global view of the A2A AR (yellow) in complex with ligand NECA (magenta). The receptor helix (H) numbers are indicated in roman numbers.

The ZMA241385 and NECA ligands have a common triazolo scaffold, and their reported experimental dissociation rate constants are 0.0252/min and 0.0510/min, respectively, at 294.15 K, which correspond to experimental residence times (RT^exp^) of 39.68 ± 7.56 min and 19.61 ± 3.69 min, respectively^32^. Before performing the SMD simulations, long conventional all-atomistic MD simulations of 500 ns were performed for both complexes to check whether the ligands unbind within this timescale. Unbinding events were not observed within this 500 ns timescale, which makes these systems ideal for performing SMD simulations to accelerate ligand unbinding. The SMD simulations were performed in a series of slow to fast unbinding simulations with pulling velocities ranging from 0.0001 to 0.0004 nm/ps, 0.0006 nm/ps, 0.0008 nm/ps, and 0.0010 nm/ps and a fixed spring force constant of 600 kJ/mol/nm^2^. Figure 3 shows the unbinding of ZMA241385 and NECA ligands with a pulling velocity of 0.0010 nm/ps along the Z-axis of the lipid membrane that is directed toward the extracellular loops of the protein receptor.

The starting structure for the SMD simulations was the final snapshot of a 100 ns production run in the NPT ensemble. The structural stability of the complexes during this 100 ns simulation was examined through the root mean square deviation (RMSD), residue-wise root mean square fluctuation (RMSF) and radius of gyration (*R*_G_) (Supplementary Information, Table S1 and Figs. S1–S3). The average RMSD values for the protein receptors were found to be 0*.*21 ± 0*.*02 nm and 0*.*21 ± 0*.*04 nm for the ZMA241385 and NECA ligand–receptor complexes, respectively, while the average *R*_G_ values for the protein receptors were estimated as 2*.*17 ± 0*.*01 nm and 2*.*21 ± 0*.*01 nm, respectively.

**Figure 4:**
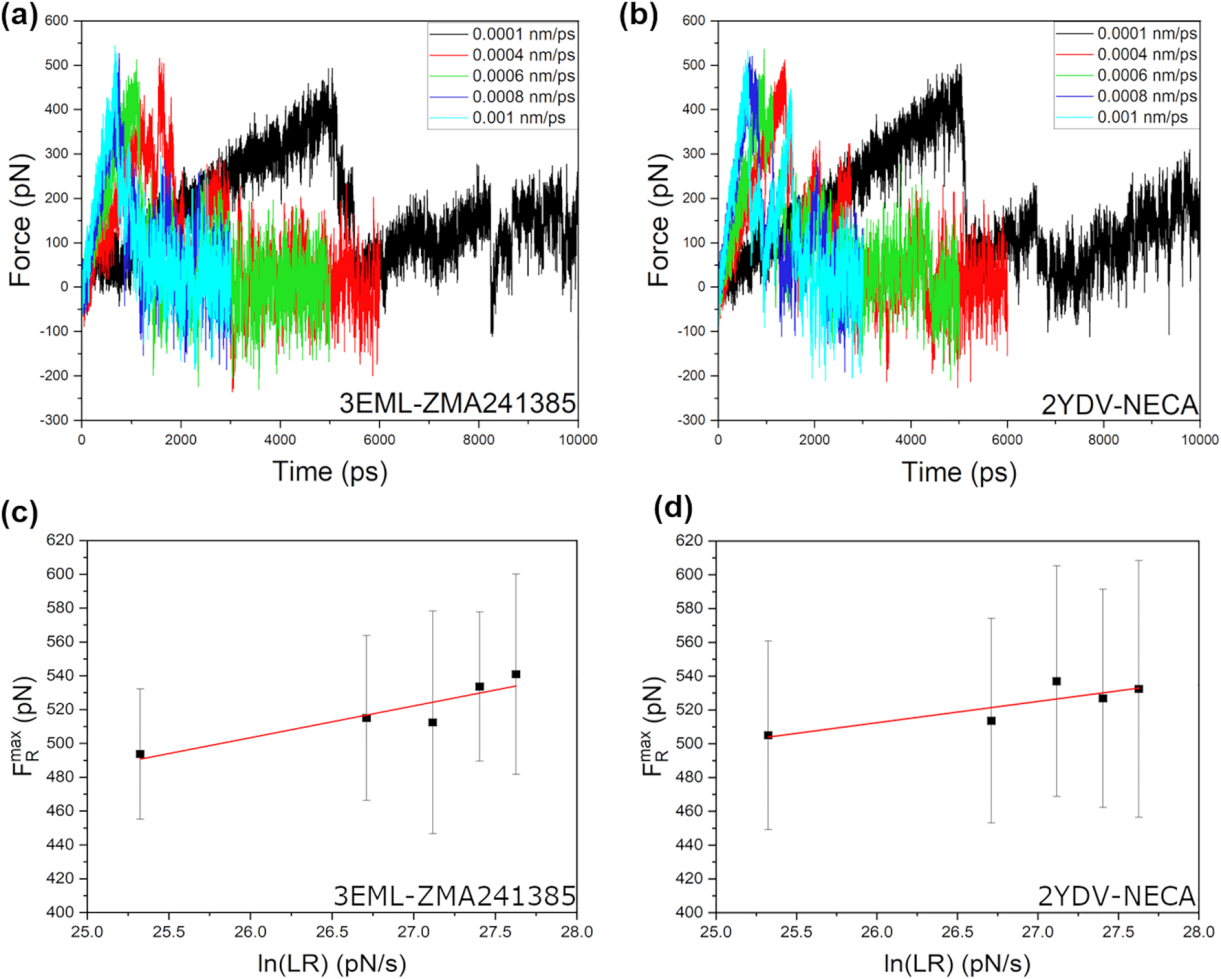
Representative unbinding force profiles of one randomly selected replica out of 41 replica SMD simulations at each of the pulling velocities for (**a**) ZMA241385 and (**b**) NECA ligand-receptor complexes. Linear least-square regression of the average maximum unbinding forces (*F*_*R*_^max^) versus natural logarithms of the LR calculated from the SMD simulations of (**c**) ZMA241385 and (**d**) NECA ligand-receptor systems. Standard deviations for the average *F*_*R*_^max^ values are shown as black error bars.

The unbinding force (*F*_*R*_) profiles during the SMD simulations were monitored for 41 replica simulations at each of the pulling velocities (Supplementary Information, Figs. S5–S14). In Fig. 4a and b, representative *F*_*R*_ profiles from one of the 41 replica simulations at each of the pulling velocities are shown. Upon careful monitoring, all 41 *F*_*R*_ profiles (Supplementary Information, Figs. S5–S14) were found to show similar observations. For a simpler understanding, the average of the maximum unbinding forces (*F*_*R*_^max^) calculated from the 41 replica simulations at each of the pulling velocities is summarized in Table 1. We also extracted the SMD simulation times (T^max^) that correspond to the *F*_*R*_^max^ values, and the average of T^max^ is reported in Table 1. Interestingly, the average T^max^ value at each of the pulling velocities was found to be higher for the ZMA241385 ligand than for the NECA ligand. This is quite reasonable considering that the RT^exp^ of the ZMA241385 ligand was reported to be higher than that of the NECA ligand (RT^exp^ = 39.68 ± 7.56 min and RT^exp^ = 19.61 ± 3.69 min for ZMA241385 and NECA, respectively, at 294.15 K)^32^. Additionally, the gradual decrease in the average T^max^ values with the gradual increase in the pulling velocity (Table 1) indicates that the choice of our SMD simulation parameters, i.e., the spring force constant (*k*) and the pulling velocity (*v*), were reasonable. We iteratively increased the number of replica simulations while monitoring the *T*^max^ values at different pulling velocities. We ended up with 41 replicas when we found that the *T*^max^ values gradually decreased with increasing pulling velocity. Comparatively lower standard deviations for the average T^max^ values at higher LR values indicate smooth ligand–receptor interactions along the unbinding pathway at higher LR values (Table 1).

**Table 1:** Loading rate (LR), average maximum unbinding force (*F*_*R*_^max^), average maximum time (T^max^) and average maximum distance (D_COM-COM_^max^) where COM of ligand was pulled from the COM of protein residues within 5 Å of ligand at each of the pulling velocities (*v*) from the SMD simulations of ZMA241385 and NECA ligand-receptor complexes.

*v* (nm/ps)	N	PDB id	Ligand	ln(LR) (pN/s)	*F*_*R*_^max^ (pN)	*t*-test	T^max^ (ps)	*t*-test	D_COM-COM_^max^ (nm)
0.0001	41	3EML	ZMA241385	25.32	493.72 ± 38.55	P(0.2760) > 0.05	5543.11 ± 751.72	P(0.0022) < 0.05	0.47 ± 0.12
		2YDV	NECA		505.05 ± 55.78		5050.15 ± 652.31		0.38 ± 0.17
0.0004	41	3EML	ZMA241385	26.71	514.99 ± 48.76	P(0.9091) > 0.05	1484.75 ± 203.58	P(0.0001) < 0.05	0.46 ± 0.11
		2YDV	NECA		513.60 ± 60.51		1312.75 ± 174.67		0.34 ± 0.12
0.0006	41	3EML	ZMA241385	27.12	512.40 ± 65.84	P(0.1006) > 0.05	1029.35 ± 170.42	P(0.8379) > 0.05	0.49 ± 0.12
		2YDV	NECA		538.82 ± 68.26		1017.53 ± 327.11		0.48 ± 0.17
0.0008	41	3EML	ZMA241385	27.40	533.64 ± 44.08	P(0.5796) > 0.05	793.23 ± 90.07	P(0.0001) < 0.05	0.45 ± 0.10
		2YDV	NECA		526.74 ± 64.54		698.75 ± 101.76		0.37 ± 0.16
0.0010	41	3EML	ZMA241385	27.63	540.93 ± 59.16	P(0.5749) > 0.05	634.05 ± 88.46	P(0.1501) > 0.05	0.37 ± 0.06
		2YDV	NECA		532.02 ± 75.99		601.89 ± 110.70		0.42 ± 0.14

The LR are reported as their natural logarithms. The LRs at different *v* were calculated by multiplying the spring force constant (*k*), 600 kJ/mol/nm^2^, and the velocities (*v*), 0.0001 nm/ps, 0.0004 nm/ps, 0.0006 nm/ps, 0.0008 nm/ps and, 0.0010 nm/ps. The average of maximum unbinding forces (*F*_*R*_^max^) and the corresponding average maximum times (T^max^) were estimated from the maximum forces and corresponding maximum times at individual replica simulations out of 41 replicas. Similarly average maximum distances (D_COM-COM_^max^) between the COM of the ligand heavy-atoms and the COM of the binding pocket residues corresponds to maximum times at which the maximum forces were recorded for each replica of the system. Results from unpaired *t*-test for the ligand-pair at a given *v* for both the *F*_*R*_^max^ and T^max^ at a confidence interval of 95% and the number of replicas (N) are listed.

Notably, the average *F*_*R*_^max^ values showed mixed observations; no specific trends were observed in relation to the LR values. Additionally, high standard deviations were observed for the average *F*_*R*_^max^. These observations could be associated with the stochastic nature of the individual ligand–receptor unbinding events inside the binding pocket and strongly correlated with the SMD simulation parameters, such as the pulling velocity and spring force constant. This observation can also be attributed to the reassignment of random velocities at 303*.*15 K at the start of each of the 41 replica SMD simulations at a particular LR. The initial velocity reassignment ensures random sampling of the initial conformational space for each of the replica simulations while pulling the ligand along the Z-axis. The *F*_*R*_^max^ values have been reported to be sensitive to subtle differences in experimental methods and the initial conformation of the ligand–receptor complex in SMD simulations^29^. Walton et al. found that for SMD simulations of biotin-streptavidin rupture, the unbinding force may vary by ∼ 20% with the initial configuration of the complex^29^.

We carried out an unpaired *t*-test for the ligand pair at a given pulling velocity for both the average maximum unbinding force (*F*_*R*_^max^) and average maximum time (*T*^max^) (Table 1). From the results of the *t*-test, based on the conventional criteria, the difference for the *F*_*R*_^max^ at any given pulling velocity was found to be not statistically significant. Interestingly, for *T*^max^, at pulling velocities of 0.0001 nm/ps, 0.0004 nm/ps, and 0.0008 nm/ps, the differences were found to be significantly different. These results suggest that obtaining statistically significant differences between both the *F*_*R*_^max^ and *T*^max^ values of the two ligands at a given pulling velocity are challenging and are the limitations of ligand unbinding rare-event simulations using SMD approach, when we compare two ligands at each of the pulling velocities. However, we would like to highlight that the main objective of this work is to predict the absolute RT of individual ligands and not to predict the relative RTs. As a secondary outcome of the absolute RT of a given ligand, qualitative ranking of different ligands can be carried out. Therefore, considering the trade-off between the computational cost of performing the simulations in multiple replicas and the reliability of the prediction, we relied only on monitoring of the *T*^max^ values at different pulling velocities for a given ligand in selecting the number of replica simulations.

**Figure 5:**
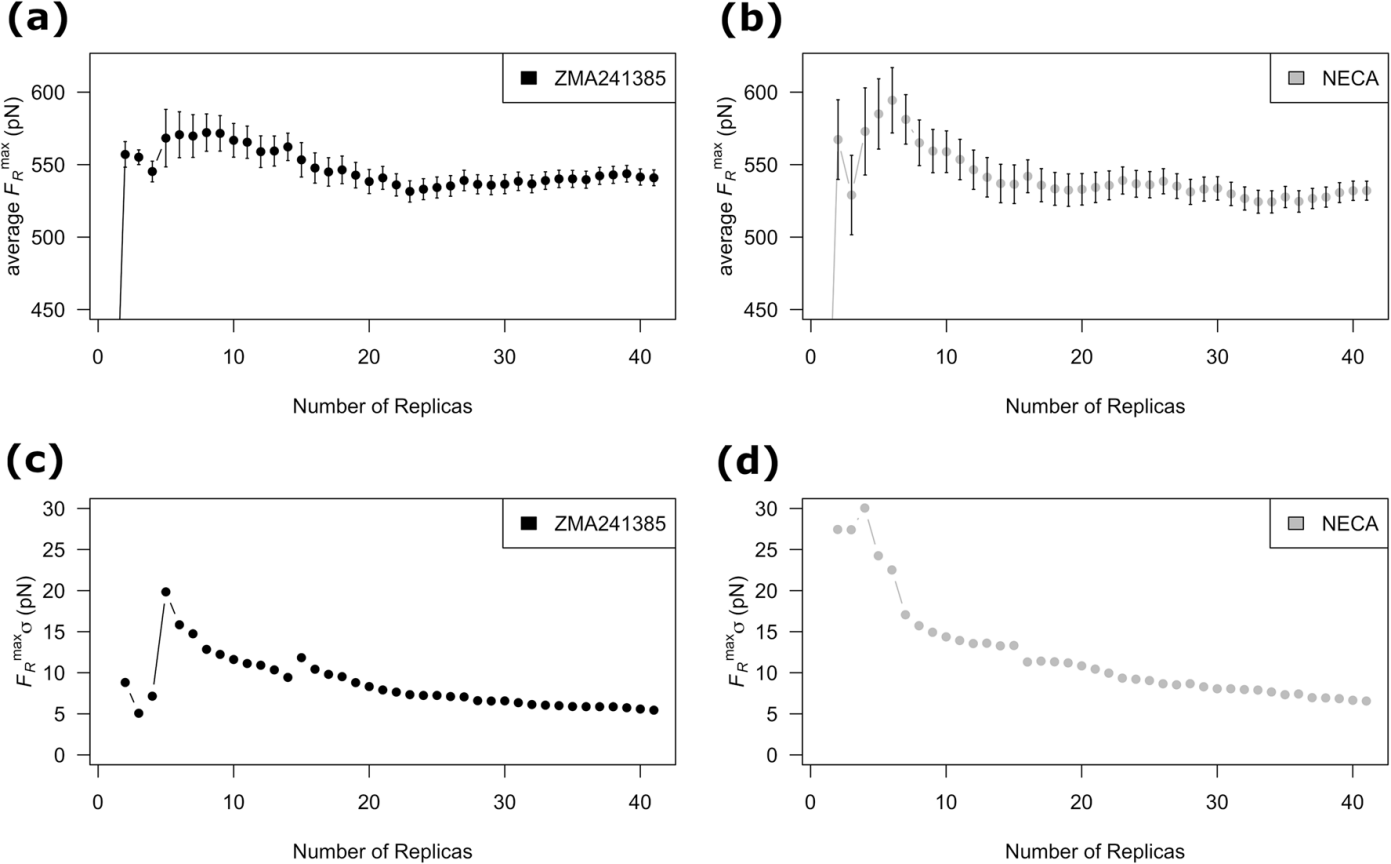
Variation of the bootstrap statistics, (**a**) and (**b**), the average maximum unbinding force (*F*_*R*_^max^) and, (**c**) and (**d**), standard deviation (*F*_*R*_^max^ σ) are shown as a function of number of replicas for the ligands ZMA241385 (black) and NECA (gray). The representative *F*_*R*_^max^ values used for the bootstrapping analysis were extracted from the SMD simulations at a pulling velocity of 0.0010 nm/ps. The error bars for the, (**a**) and (**b**), average *F*_*R*_^max^ values are the *F*_*R*_^max^ σ values.

In this regard, the bootstrapping analysis on the *F*_*R*_^max^ values with increasing number of replicas provides additional confidence (Fig. 5). As shown in Fig. 5a and b, increase in the number of replicas results in significant change in the average *F*_*R*_^max^ values. This observation indicates that average *F*_*R*_^max^ values are sensitive to the choice of number of replicas. However, the standard deviation (*F*_*R*_^max^ σ) at each of bootstrap steps for the average of the *F*_*R*_^max^ is gradually decreasing (Fig. 5c and d). The *F*_*R*_^max^ σ values for both the ligands were found to be converging around 40 replicas. Potterton et al. followed similar bootstrapping approach to decide on the choice of optimal number of replicas for the prediction of relative RTs of A2A AR binders. They ended up with selecting 10 replicas after performing 30 replica simulations for an effective trade-off between minimizing the error, stabilization of average property and the computational cost^33^.

Additionally, we generated average unbinding force profiles with the objective of selecting the number of replica simulations for both ligands at different LRs. To generate these profiles, averaging of the unbinding forces was carried out every 0.1 ps along the SMD trajectories of the 41 replica simulations (Supplementary Information, Fig. S4). For each of the two ligands, along the simulation trajectories, the computational times corresponding to the maxima of the average unbinding force profiles were found to gradually decrease with increasing pulling velocity. The characteristics of the average unbinding force profiles were found to be similar to the individual profiles (Fig. 4a, b and Supplementary Information, Figs. S5–S14 and Table S2). These observations suggest that 41 replicas were sufficient for the generation of a statistically reliable dataset for the employment of the Bell-Evans model.

Linear least-square regression of the average maximum unbinding force *F*_*R*_^max^ (pN) versus the natural logarithm of the LR (pN/s) (Table 1) was carried out (Fig. 4c, d). The goodness of the linear regressions was measured by the R-squared (*R*^2^) values, which were found to be 0.84 and 0.74 for the ZMA241385 and NECA ligand–receptor systems, respectively (Table 2). To estimate the dissociation rate constant (*k*_*off*_^SMD^) from our SMD simulations, the Bell-Evans model was applied as described in the “Methods” section. The predicted *k*_*off*_^SMD^ values were 0*.*04/s and 1*.*17/s for the ligands ZMA241385 and NECA, respectively (Table 2). The ligand absolute RTs estimated from the predicted *k*_*off*_^SMD^ values were found to be 25*.*00 s and 0*.*86 s for the ligands ZMA241385 and NECA, respectively. In experimental studies, the ligand RT of ZMA241385 was also determined to be higher than that of NECA (RT^exp^ = 2380.80 ± 453.60 s and 1176.60 ± 221.40 s for ZMA241385 and NECA, respectively, at 294.15 K)^32^ (Table 2). However, compared to our prediction, the RTs reported from experimental studies were ∼ 95-fold and ∼ 1368-fold longer for the ZMA241385 and NECA ligands, respectively.

**Table 2:** Estimated statistics from the linear least-square regression of the average maximum unbinding forces (*F*_*R*_^max^) versus natural logarithms of the LR calculated from the SMD simulations of (a) ZMA241385 and (b) NECA ligand-receptor systems, the predicted dissociation rate constant (*k*_*off*_^SMD^) and the corresponding residence time (RT^SMD^), and the experimental residence time^32^ (RT^exp^) are reported.

PDB id	Ligand	y-intercept	Slope (*m*)	x-intercept (*b*)	*R*^2^	*k*_off_^SMD^ (s^−1^)	RT^SMD^ (s)	RT^exp^ (s)
3EML	ZMA241385	14.90	18.79	0.79	0.84	0.04	25.00	2380.80 ± 453.60
2YDV	NECA	184.44	12.63	14.61	0.74	1.16	0.86	1176.60 ± 221.40

### Ligand binding affinity from conventional molecular dynamics (MD) simulations

To better understand the energetics of ligand–receptor interactions inside the binding pocket of the A2A AR, the ligand binding affinities were estimated by calculating the MMPBSA-based ligand binding energy (*E*_*bind*_) and its components: the van der Waals (vdW) interaction energy (*E*_*int*_^vdW^), electrostatic interaction energy (*E*_*int*_^elec^), polar solvation energy (*E*^polar^), and nonpolar solvation energy (*E*^non−polar^) (Table 3). The ZMA241385 ligand was found to interact more favorably with the A2A AR, with a significantly lower binding energy of − 87*.*42 kJ/mol compared to the NECA ligand, the binding energy of which was estimated as − 45*.*28 kJ/mol. For both ligands, *E*_*int*_^vdW^ < *E*_*int*_^elec^ < *E*^non−polar^ < *E*^polar^, indicating that the major contributions to the ligand binding energies are from the net nonbonded interaction energies (*E*_*int*_^net^) that come from the summation of the *E*_*int*_^vdW^ and *E*_*int*_^elec^ components. The *E*_*int*_^net^ values for the ZMA241385 and NECA ligands were estimated as − 178*.*53 kJ/mol and − 144*.*69 kJ/mol, respectively. Notably, the vdW interaction energy components of the net nonbonded interaction energies were found to contribute more to the stabilization of both ligands in the binding pocket. Other than the nonbonded interaction energies, stabilization of ligand binding through the nonpolar solvation energies was also observed for both ligands.

**Table 3:** Ligand-receptor binding energy (*E*_*bind*_^total^) and its components, the vdW interaction energy (*E*_*int*_^vdW^), the electrostatic interaction energy (*E*_*int*_^elec^), the polar solvation energy (*E*^polar^) and, the non-polar solvation energy (*E*^non−polar^).

PDB id	Ligand	*E*_*int*_^vdW^ (kJ/mol)	*E*_*int*_^elec^ (kJ/mol)	*E*^polar^ (kJ/mol)	*E*^non−polar^ (kJ/mol)	*E*_*bind*_^total^ (kJ/mol)
3EML	ZMA241385	− 142.27 ± 10.66	− 36.26 ± 9.99	108.47 ± 15.92	− 17.36 ± 0.97	− 87.42 ± 11.87
2YDV	NECA	− 121.89 ± 8.62	− 22.80 ± 8.21	114.90 ± 11.13	− 15.50 ± 0.84	− 45.29 ± 10.26

The MMPBSA-based calculations were carried out on the last 10 ns of the 100 ns conventional MD simulations performed prior to the SMD simulations. The *E*^non−polar^ was estimated by using the non-polar solvation model of solvent accessible surface area (SASA). The contributions from the net non-bonded interaction energies (*E*_*int*_^net^), those were calculated in vacuum, to the binding energies were decomposed into *E*_*int*_^vdW^ and *E*_*int*_^elec^.

### Identifying energetic hotspots within the A2A adenosine receptor

The MMPBSA-based ligand binding energies (*E*_*bind*_^total^) were further decomposed at the individual residue level (*E*_*bind*_^res^). In Table 4, we list the top 12 residues that contributed <  − 1*.*00 kJ/mol to the total ligand binding energies. In the case of the A2A AR bound to the ligand ZMA241385, except for the residues ASP170 and GLU13, all the residues contributed most favorably to the total ligand binding energy through the molecular mechanics net nonbonded interaction energy (*E*^MM^) component, which can be defined as the summation of the vdW and electrostatic interaction energies. *E*^MM^ was also found to be the major component contributing to the ligand binding energy of NECA. For both the ZMA241385 and NECA ligands, the residue ASP170 was found to favorably contribute to the ligand binding energies through the polar solvation energy (*E*^polar^) component.

**Table 4:** Top 12 residues of the A2A AR those contributed to the total ligand binding energies (*E*_*bind*_^total^) by <  − 1*.*00 kJ/mol.

PDB id	Ligand	Residue	*E*_*bind*_^res^ (kJ/mol)	E^MM^ (kJ/mol)	E^polar^ (kJ/mol)	E^non−polar^ (kJ/mol)
3EML	ZMA241385	**PHE168**	− **8.25 ± 0.15**	**− 11.72 ± 0.16**	**4.54 ± 0.12**	**− 1.07 ± 0.02**
		**LEU249**	− **7.70 ± 0.11**	**− 8.81 ± 0.11**	**1.72 ± 0.07**	**− 0.61 ± 0.02**
		**MET270**	− **6.26 ± 0.18**	**− 6.91 ± 0.21**	**1.50 ± 0.08**	**− 0.85 ± 0.03**
		**ILE274**	− **4.19 ± 0.11**	**− 4.59 ± 0.12**	**1.09 ± 0.03**	**− 0.69 ± 0.02**
		**MET177**	− **2.98 ± 0.08**	**− 3.74 ± 0.08**	**0.91 ± 0.05**	**− 0.15 ± 0.01**
		**TRP246**	− **2.77 ± 0.11**	**− 3.14 ± 0.11**	**0.78 ± 0.05**	**− 0.41 ± 0.01**
		ASP170	− 2.72 ± 0.10	− 0.45 ± 0.07	− 2.27 ± 0.08	0.00 ± 0.00
		GLU13	− 2.42 ± 0.11	0.67 ± 0.07	− 3.09 ± 0.11	0.00 ± 0.00
		TYR271	− 1.97 ± 0.19	− 3.52 ± 0.16	2.10 ± 0.13	− 0.55 ± 0.02
		LEU267	− 1.81 ± 0.09	− 1.36 ± 0.10	− 0.16 ± 0.04	− 0.29 ± 0.02
		**VAL84**	**− 1.79 ± 0.08**	**− 2.67 ± 0.09**	**1.15 ± 0.04**	**− 0.27 ± 0.01**
		**LEU85**	**− 1.65 ± 0.10**	**− 2.17 ± 0.10**	**0.75 ± 0.02**	**− 0.23 ± 0.01**
2YDV	NECA	**PHE168**	**− 8.15 ± 0.19**	**− 12.26 ± 0.19**	**5.06 ± 0.14**	**− 0.95 ± 0.02**
		**LEU85**	**− 3.45 ± 0.10**	**− 6.24 ± 0.14**	**3.08 ± 0.10**	**− 0.29 ± 0.01**
		**VAL84**	**− 3.22 ± 0.10**	**− 5.79 ± 0.12**	**2.98 ± 0.06**	**− 0.41 ± 0.02**
		**TRP246**	**− 3.11 ± 0.11**	**− 3.72 ± 0.14**	**1.32 ± 0.08**	**− 0.71 ± 0.03**
		**MET177**	**− 2.32 ± 0.07**	**− 3.16 ± 0.09**	**1.21 ± 0.05**	**− 0.37 ± 0.02**
		ALA63	− 1.95 ± 0.12	− 3.71 ± 0.15	2.21 ± 0.10	− 0.45 ± 0.02
		ASP52	− 1.69 ± 0.12	− 2.39 ± 0.13	0.70 ± 0.07	0.00 ± 0.00
		ILE60	− 1.25 ± 0.06	− 0.57 ± 0.05	− 0.51 ± 0.02	− 0.17 ± 0.01
		CYS185	− 1.16 ± 0.11	− 1.73 ± 0.08	0.73 ± 0.06	− 0.16 ± 0.01
		ALA89	− 1.11 ± 0.05	− 1.66 ± 0.05	0.65 ± 0.03	− 0.10 ± 0.01
		ILE66	− 1.10 ± 0.06	− 1.73 ± 0.06	0.72 ± 0.04	− 0.09 ± 0.01
		ASP170	− 1.01 ± 0.04	0.77 ± 0.05	− 1.78 ± 0.06	0.00 ± 0.00

Decomposition of the residue-wise contributions (*E*_*bind*_^res^) into the molecular mechanics net non-bonded interaction energies (*E*^MM^), polar solvation energies (*E*^polar^) and, non-polar solvation energies (*E*^non−polar^) are summarized. Residues within 5 Å of the ligands ZMA241385 and NECA in the binding pocket of the crystal structure of the A2A AR-ligand complex (PDB ids: 3EML and 2YDV, respectively) and that contributed favorably to the binding energies of both the ligands by <  − 1*.*00 kJ/mol are shown in bold.

**Figure 6:**
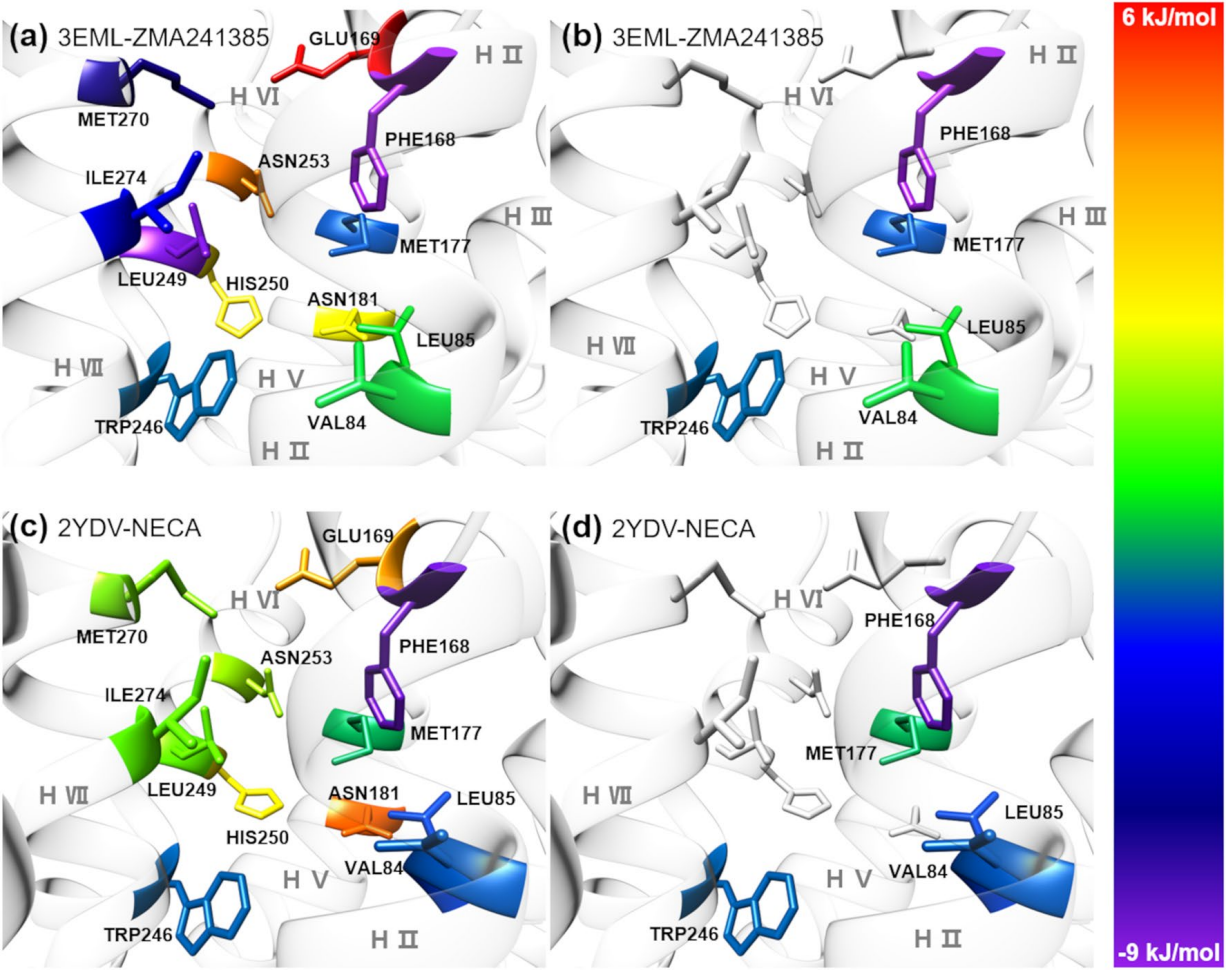
Decomposition of the MMPBSA-based total ligand binding energies (*E*_*bind*_^total^) at the individual residue level (*E*_*bind*_^res^). 12 Common residues within 5 Å of the ligands (**a**) ZMA241385 and (**c**) NECA in the binding pocket of the crystal structure of the A2A AR-ligand complex (PDB ids: 3EML and 2YDV, respectively). 5 structural hotspot residues which are common and contribute favorably to the binding energies of both the ligands (**b**) ZMA241385 and (**d**) NECA by <  − 1*.*00 kJ/mol and are present within 5 Å of the ligands inside the binding pocket of A2A AR. The residues are colored based on their contribution, *E*_*bind*_^res^, reported in Supplementary Information, Table S3, to the *E*_*bind*_^total^ (see colour bar).

With the objective of identifying structural hotspots within the A2A AR, we list 12 common protein residues in the binding pocket of the A2A AR that are within 5 Å of the ligands ZMA241385 and NECA (Supplementary Information, Table S3). Interestingly, all 12 residues in proximity to the ligands did not favorably contribute to the ligand binding energies (Fig. 6a, c; Supplementary Information, Table S3). Based on the favorable contributions to the ligand binding energy of ZMA241385, the order of protein residues within 5 Å of the ligand is PHE168 > LEU249 > MET270 > ILE274 > MET177 > TRP246 > VAL84 > LEU85, whereas that for the NECA system is PHE168 > LEU85 > VAL84 > TRP246 > MET177. Based on these results, we selected the hotspots as VAL84, LEU85, PHE168, MET177 and TRP246, which are the common residues that contributed favorably to the binding energies of both the ligands ZMA241385 and NECA by <  − 1.00 kJ/mol and are present within 5 Å of the ligands inside the binding pocket of the A2A AR (Table 4; Fig. 6b, d). Interestingly, residues LEU249, MET270 and ILE274 were found to favorably contribute to the binding energy of ZMA241385 by − 7.70 kJ/mol, − 6.26 kJ/mol and − 4.19 kJ/mol, respectively, whereas the same residues were found to contribute the least to the binding energy of NECA by − 0.53 kJ/mol, − 0.32 kJ/mol and − 0.61 kJ/mol, respectively (Supplementary Information, Table S3). Notably, GLU169, ASN181, HIS250, and ASN253 unfavorably contributed to the binding energies (*E*_*bind*_^total^) of both ligands (Supplementary Information, Table S3).

## Discussion

In this work, we have predicted the dissociation rate constants (*k*_*off*_^SMD^) and estimated the corresponding residence times (RT^SMD^) for the two ligands ZMA241385 and NECA that bind to the A2A AR of the GPCR protein family^34,35^. Our estimations of the absolute RTs were on the timescale of seconds, however, they were many folds shorter than those determined experimentally. We have tested the approach of combining the Bell-Evans model and SMD simulations for the prediction. The ZMA241385 ligand has been reported to be an A2A adenosine subtype-selective high-affinity antagonist of naturally occurring caffeine^34^. The NECA ligand, on the other hand, is an agonist of the A2A AR and contains a ribose moiety that is not found in ZMA241385^35^ (Fig. 1b, c). Although the chemically related regions of the ZMA241385 and NECA ligands have been reported to bind in a similar fashion in the binding pocket of the A2A AR, the antagonist activity of the ligand ZMA241385 was proposed to be associated with the restricted conformational freedom of helix V of the A2A AR^35^. As such, reliable prediction of the kinetic and thermodynamic parameters for these ligands would be of great interest for the development of drug candidates with extended pharmacological activity in vivo. Promisingly, this approach was found to be efficient enough to predict the absolute RTs of these ligands on the timescale of seconds.

From the binding affinity analyses of the ZMA241385 and NECA ligands in terms of their binding energies, a negative correlation was observed between the ligand binding affinities and the dissociation rate constants (*k*_*off*_), which agrees with Eq. (2). This observation confirms that stronger binding favors slower unbinding. Our results also suggest that modulating the nonbonded interaction energy between the ligands and the receptor, especially the vdW interaction energy components, would be useful for designing more potent binders. In this regard, identification of the hotspot residues believed to anchor the ligands in the binding pocket points toward a more specific direction. Among the identified hotspot residues VAL84, LEU85, PHE168, MET177 and TRP246, residue TRP246 has been reported to be in significant contact with the ZMA241385 ligand in the X-ray crystal structure^34^. Residue TRP246 has been found to contribute almost equally, by − 2.77 kJ/mol and − 3.11 kJ/mol, to the ligand binding energies (*E*_*bind*_^total^) of ZMA241385 and NECA, respectively (Table 4). The hotspot residue PHE168 that contributed the most to the *E*_*bind*_^total^ of both ligands has been reported to be in aromatic *π*-stacking interactions with both ligands in the X-ray crystal structures^34,35^ (Table 4). The other two hotspot residues VAL84 and LEU85 have been found to be in close contact with the ribose moiety of the NECA ligand in the X-ray crystal structure^35^. Comparative studies on the X-ray crystal structures of ZMA241385 and NECA ligands bound to the A2A AR suggested a 2 Å shift of helix III to accommodate the ribose moiety of the NECA ligand^35^. Interestingly, the interaction pattern between the residue VAL84 and ligands was proposed to be specific for agonists, and mutagenesis studies have indicated a major role of this hotspot residue in the activation of the receptor^35–37^. In the X-ray crystal structures, close vdW contact between the hotspot residue MET177 and the NECA ligand has been reported, whereas MET177 is in hydrophobic contact with the ZMA241385 ligand^34,35^. Most interestingly, among the 12 common residues present within 5 Å of both ligands, GLU169, HIS250, and ASN253 unfavorably contribute to the ligand binding energies (*E*_*bind*_^total^) by > 0*.*00 kJ/mol (Supplementary Information, Table S3). These three residues have been reported to be points of mutations associated with disruption of agonist and/or antagonist binding and can be referred to as anti-hotspot residues^38–40^. Another residue, ILE274, which is also reported to be an important mutation point^38–40^ and is present within 5 Å of both ligands, favorably contributes to the ligand binding energies of ZMA241385 and NECA by − 4*.*19 kJ/mol and − 0*.*61 kJ/mol (Supplementary Information, Table S3). Along this line, we predicted an additional common residue, ASN181, within 5 Å of ligands ZMA241385 and NECA that unfavorably contributes to the ligand binding energies by 0*.*99 and 4*.*58 kJ/mol, respectively, and can be considered for mutagenesis studies.

Mutation studies of the hotspot residues and chemical modification of the ZMA241385 and NECA ligands and subsequent iterative application of our approach for predicting the kinetic parameters and the computationally less expensive and easy-to-implement MMPBSA approach^31^ could be considered a general purpose pipeline for the development of better therapeutics for diseases such as Parkinson’s^41,42^, Huntington’s^43^, asthma^44^, seizures^45^, pain^46^ and many more^47^ that are associated with the adenosine class of GPCRs. Notably, from biochemical studies, a 2-phenylhydrazino derivative of ZMA241385, namely, LUF5475, showed comparable and high affinities for the A2A and A2B ARs but was found to be selective for the A2B receptor compared to the A1 and A3 receptors^48^. Atomistic studies on the thermodynamics and kinetics of LUF5475-receptor interactions have yet to be explored. However, further validation of our approach is required for different GPCRs. Mutation studies will be beneficial for rationalizing the binding energies of ligands with respect to their RTs. Specifically, application of our approach for GPCRs with mutated hotspot residues and studying the effect of mutation on the ligand RT will allow additional validation of our strategy.

SMD simulations have already been found to be efficient for predicting the RT of diverse classes of protein receptors and ligand complexes^29,33,49–51^. Wong and coworkers in their two recent consecutive studies reported the applicability of SMD simulations in identifying and designing ligands with long RTs targeting focal adenosine kinase (FAK) as a test system^52,53^. Their most recent work reported a strong correlation between the computational ligand unbinding times for 14 ligands and their experimental dissociation rate constant (*k*_*off*_) values^52^. The nonpolar ligand–receptor interaction energy components and the distances of the nonpolar receptor residues from the ligands were found to be strongly correlated to the experimental *k*_*off*_ values. Although their regression model has been proposed to be useful in identifying chemically related ligands with desired dissociation rate constants, one ligand appeared as an outlier in the regression analysis, and the model was trained for the FAK receptor only. Additionally, Wong and coworkers never attempted to predict the dissociation rate constants (*k*_*off*_) and corresponding RTs at the level of individual ligands using the data extracted from the SMD simulations in multiple replicas. The general applicability of our approach to any chemically unrelated ligand targeting any receptor molecule would require additional large-scale validation, which we are currently working on. Interestingly, application of the Bell-Evans model has already been proposed to be very promising in predicting the kinetic parameters for unbinding of streptavidin from biotin using SMD simulations^29^.

Unlike the selectively scaled molecular dynamics (ssMD) approach^54^, a limitation of our approach is associated with the positional restraints applied on the backbone heavy atoms of the receptor during the SMD simulations to prevent the receptor from drifting along the pulling direction. Another significant limitation of our approach is the prediction of absolute RTs on the timescale of seconds, which were found to be ∼ 95-fold and ∼ 1368-fold shorter for the ZMA241385 and NECA ligands, respectively, than those determined experimentally (Table 2). Similar differences have already been reported for the ssMD approach, in which a large discrepancy on the order of ∼10^4^ was observed between the predicted unbinding times at the unscaled potential and experimentally measured RTs^54^. In this regard, an enhanced sampling approach, namely, *τ*-random accelerated molecular dynamics (*τ*-RAMD), has been reported to be very promising in predicting the ligand RT^55–58^. Similar to the SMD simulation approach, acceleration of ligand unbinding can be achieved in the *τ*-RAMD method by applying an additional randomly oriented force in an adaptive manner to the ligand center of mass. The *τ*-RAMD approach has been rigorously tested for membrane-embedded GPCRs, specifically the *β*2-adrenergic receptor (*β*2AR) and muscarinic acetylcholine receptor M2 (mAChR M2), in their complexes with orthosteric and allosteric compounds^58^. The results from the *τ*-RAMD approach were found to be in robust agreement with those observed in experimental studies^58^. This approach was reported to correctly rank four ligands based on their predicted relative RTs^58^. In the ssMD approach, a short distance cutoff of 10 Å was chosen for the native contacts between the receptor and the ligand to ensure that ligand unbinding was a first-order process^54^. In our work, to employ the Bell-Evans model on SMD data, maximum unbinding forces (*F*_*R*_^max^) were calculated along the SMD simulation trajectories. In SMD simulation of a ligand–receptor complex in which the ligand is pulled from the binding pocket of the receptor using a user-specified LR, the maximum unbinding force corresponds to the maximum force required to rupture all the native contacts between the ligand and the receptor for initial unbinding but not for complete unbinding^59^. In our work, for many of the unbinding force (*F*_*R*_) profiles, multiple peaks were observed (Supplementary Information, Figs. S5–S14). The initial maximum peaks correspond to the rupture of native contacts, and the following peaks can be assigned to the energy barriers associated with the short-lived interactions between the ligand and the receptor along the unbinding pathway. Interestingly, the average maximum distances (D_COM-COM_^max^) between the center of mass (COM) of the ligand heavy atoms and the COM of the binding pocket were found to be as short as < 5 Å, ranging from 3.7 to 4.9 Å for the 3EML system and 3.4–4.8 Å for the 2YDV system (Table 1). This observation indicates that the absolute RT estimated from the predicted dissociation rate constant (*k*_*off*_) corresponds to the initial unbinding of the ligands and provides a plausible explanation for the shorter timescale of the estimated RT compared to the experimentally determined RT. Additionally, the observed differences in the timescale may be associated with the choice of large LR in our study, which is in the range of 25–27 pN/s (as estimated from the natural logarithm of the LR), whereas the experimental LR typically falls below 8 pN/s, as measured for the biotin-streptavidin complex^29^. Notably, Walton et al. proposed a correction to the unbinding force (*F*_*R*_) of 1*/*2*kx*_*b*_ to eliminate the dependence of *k*_*off*_ and *x*_*b*_ on the spring force constant (*k*) and reported closer agreement between the kinetic parameters predicted from SMD simulations and those measured from experiments. This correction approach is beyond the scope of our study because we have performed SMD simulations when the spring force constant was fixed at a certain value and only the pulling velocity was varied^29^. Therefore, for constant velocity (fixed spring force constant) SMD simulations, appropriate choice of lower pulling velocities, accounting for lower LR, would be more appropriate for the prediction of the equilibrium dissociation rate constant (*k*_*off*_) from non-equilibrium SMD simulations. A lower pulling velocity would require a longer simulation time for the initial unbinding, as can be clearly seen from the unbinding force profiles at different pulling velocities (Supplementary Information, Figs. S5–S14 and Table 1). As such, our choices of pulling velocity and spring force constant were found to be reasonable in establishing a trade-off between the speed and accuracy in predicting the dissociation rate constants (*k*_*off*_) for the ZMA241385 and NECA ligands targeting the A2A AR and should be applicable to other systems as well. As a future scope of improving our approach for its wide applicability, we are currently working on extensive validation of our approach on a diverse variety of receptor–ligand systems. The limitations associated with our MMPBSA protocol, and the force field inaccuracies also cannot be ignored^60^. For simplicity and to reduce the computational cost, we disregarded the membrane in our MMPBSA calculations of ligand binding energies considering that the protein receptors, under study, experience similar membrane environments. However, inclusion of membranes implicitly or explicitly in the calculations may have impacted on the environment of the individual protein receptors and subsequently on the binding energies of the ligands to the proteins^61^. In traditional MMPBSA calculations, the polar solvation energy (*E*^polar^) is calculated considering a continuum of high dielectric constant around the molecular system of interest. Our protein–ligand systems in the presence of membranes may not have replicated such environment appropriately. Regarding the ligand force field parameters, we are planning to perform rigorous parameterization to improve the accuracy of our prediction. Allowing flexibility in the extracellular loops during our SMD simulations would be also interesting to study for capturing the intermediate states and exploring the variability in entrance and/or exit routes.

## Methods

### Computational details

The molecular mechanics calculations and molecular dynamics (MD) simulations were performed using the GROMACS 2019^62,63^ simulation package. The CHARMM36 protein force field^64^ was used for the human A2A adenosine receptor, whereas for the 1-palmitoyl-2-oleoyl-sn-glycero-3-phosphocholine (POPC) lipid bilayer, the CHARMM36 lipid force field^65^ was used. The CHARMM general force field (CGenFF)^66^ was used for parameterization of the ZM241385 and NECA ligands. The simulation systems and the initial GROMACS simulation scripts were generated using the CHARMM-GUI webserver^67–69^.

### System preparation

The primary structures for the simulations of human A2A adenosine receptors in complex with the antagonist ligand ZMA241385 and agonist ligand NECA were obtained from the protein data bank (PDB) via the PDB accession numbers 3EML^34^ and 2YDV^35^ (Fig. 1b, c). The primary structures were loaded into the CHARMM-GUI webserver^67–69^, where the missing residues were built and the whole system was oriented along the principal Z-axis. The crystallographic water molecules were retained. The fully assembled system from the CHARMM-GUI web server was aligned, utilizing PyMOL molecular graphics software, with the respective coordinates downloaded from the OPM server^70^. The aligned systems for both structures were trimmed by shortening the C-terminal of the receptor for PDB id: 2YDV and by deleting the lysozyme part of the receptor for PDB id: 3EML to obtain a uniform receptor. The modified systems were then further processed through the CHARMM-GUI web server to obtain the final assembled systems with disulfide bonds. The ligand–receptor systems embedded inside the POPC lipid bilayer were built using the CHARMM-GUI membrane builder^68^ (Fig. 1a). The systems were solvated with TIP3P^71^ water molecules. The solvated systems were neutralized using K^+^ ions. Excess KCl salt was added to obtain a final salt concentration of 0.15 M. The initial system parameters of both systems are summarized in Supplementary Information, Table S4.

### Conventional molecular dynamics (MD) simulation

The initial systems were energy minimized by employing the steepest descent algorithm implemented in GROMACS using an energy threshold of 10 kJ/mol/nm. After minimization, equilibration simulations were performed for 25 ns in the NVT ensemble. Temperature and pressure coupling during the equilibration runs were applied using the Berendsen algorithm^72^. Restraints on heavy atoms of the lipids, protein and ligand backbones and side chains were applied during the equilibration run. After equilibration, 100 ns production runs in an isothermal-isobaric ensemble (NPT) were carried out for both systems, in which all the restraints were lifted. During the production run, temperature coupling was applied using the Nose–Hoover algorithm^73,74^, whereas the Parrinello-Rahman algorithm^75^ was used for the pressure coupling. A temperature coupling constant of 1.0 ps and a pressure coupling constant of 5.0 ps were employed. Throughout the production run, the pressure was kept constant at 1 atm. An integration timestep of 2 fs was used for both the equilibration and production runs. Simulations were performed at a temperature of 303.15 K. Nonbonded interactions were truncated at 1.2 nm with the Verlet cutoff scheme^76^. Long-range electrostatic interactions were modeled by the particle mesh Ewald (PME) technique^77^. Hydrogen bonds were constrained using the LINCS^78^ algorithm. Simulations were subjected to periodic boundary conditions in a semi-isotropic environment. The system coordinates were saved every 50,000 steps, which corresponded to 100 ps during the production run. Additionally, an independent 500 ns production run simulation was performed for both systems by regenerating the velocities after equilibration.

### Steered molecular dynamics (SMD) simulation

The velocities after the 100 ns production run were discarded. New random velocities were assigned to the final coordinates of the 100 ns run in 41 replicas, and normal simulations were continued for 50 ps before applying the pulling velocity. The time evolutions of the temperature and kinetic energy for the 50 ps run from one representative simulation out of 41 replicas are presented in the Supplementary Information as Fig. S15. The time evolutions clearly indicate the equilibration of newly assigned velocities within 50 ps. At the end of the 50 ps equilibration simulations, SMD simulations were initiated by pulling the center of mass (COM) of the heavy atoms of the ligands from the COM of the residues of the A2A adenosine receptor within 5 Å of the ligands. The pulling of the ligands was performed along the Z-axis of the lipid membrane, directed toward the extracellular loops of the protein. We applied pulling velocities of 0.0001 nm/ps, 0.0004 nm/ps, 0.0006 nm/ps and 0.0008 nm/ps and 0.0010 nm/ps and a spring force constant of 600 kJ/mol/nm^2^ during our SMD simulations. Regarding the choice of the spring force constant, we performed many trials using 200 and 400 kJ/mol/nm^2^ for the lowest pulling velocity simulations. We did not observe many unbinding events within 10 ns of the simulations for the spring force constant values of 200 and 400 kJ/mol/nm^2^. Spring force constant values higher than 600 kJ/mol/nm^2^ would accelerate the ligand unbinding events but may very abruptly disrupt the receptor–ligand interaction in the binding pocket. Therefore, we selected 600 kJ/mol/nm^2^ as the spring force constant. The time lengths of the SMD simulations at each of the velocities were set to ensure complete unbinding of the ligands from the binding pocket of the A2A adenosine receptor. For pulling velocities of 0.0001 nm/ps, 0.0004 nm/ps, 0.0006 nm/ps, 0.0008 nm/ps and 0.0010 nm/ps, SMD simulations were carried out for 10 ns, 6 ns, 5 ns, 3 ns and 3 ns, respectively. A positional restraining force of 1000 kJ/mol/nm^2^ was applied on the receptor backbone heavy atoms to avoid drift of the whole receptor in the pulling direction. The coordinates were recorded every 1 ps, and the unbinding forces were written out at each 0.1 ps of the SMD trajectories.

### Choice of optimal number of replicas SMD simulations

To decide on the optimal number of replicas, the averages of the maximum unbinding forces (*F*_*R*_^max^) calculated from the replica simulations at one of the pulling velocities of 0.0010 nm/ps were monitored with increasing number of replicas. This is because *F*_*R*_^max^ is the key parameter extracted from the SMD simulations and is the basis of the calculation of the dissociation rate constant (*k*_*off*_) following Bell-Evans model as described below. To examine the effect of number of replicas on the average *F*_*R*_^max^, we carried out bootstrapping analysis^79^. In our bootstrapping analysis, the *F*_*R*_^max^ data points were resampled 10000 times. The average of the *F*_*R*_^max^ and standard deviation (*F*_*R*_^max^ σ) at each bootstrap was calculated. The *F*_*R*_^max^ σ provides an estimate of error associated to the average *F*_*R*_^max^ calculated at each bootstrap step.

### Employment of the Bell-Evans model

The dissociation rate constant (*k*_*off*_) and the energetic unbinding distance (*x*_*b*_) between the bound state and the energetic maximum for each of the ligands were predicted simply from the slope (*m*) and x-intercept (*b*) of the linear least-square regression of the average maximum unbinding force (*F*_*R*_^max^) versus the natural logarithm of the loading rate (LR), ln(LR), extracted from the SMD simulations. The LR at different velocities was calculated by multiplying the spring force constant (*k*), 600 kJ/mol/nm^2^, and the velocities (*v*), 0.0001 nm/ps, 0.0004 nm/ps, 0.0006 nm/ps, 0.0008 nm/ps and 0.0010 nm/ps. Following the Bell-Evans model (Eq. 3), the energetic distance (*x*_*b*_) between the bound state and the energetic maximum can be expressed as

4 $$x_{b} = \frac{{k_{B} T}}{m}$$

where *k*_*B*_is Boltzmann’s constant, *T* is absolute temperature, and *m* is the slope, and the dissociation rate constant (*k*_*off*_) can be expressed as

5 $$k_{off} = b\left( {\frac{{x_{b} }}{{k_{b} T}}} \right)$$

By combining Eqs. (4) and (5).

6 $$k_{off} = b\left( {\frac{{k_{B} T}}{m}} \right)\left( {\frac{1}{{k_{B} T}}} \right) = \frac{b}{m}$$

where *b* refers to the x-intercept and *m* refers to the slope of the linear least-square regression of *F*_*R*_^max^ versus ln(LR). The inverse of the *k*_*off*_ value (i.e., 1*/k*_*off*_) yields the absolute RT of the ligand.

### Trajectory analysis

Trajectories were analyzed using the analysis utilities embedded in the GROMACS package. The root mean square deviation (RMSD), root mean square fluctuation (RMSF) and radius of gyration (*R*_*G*_) were computed to confirm the structural stability of the A2A adenosine receptor–ligand complexes. The *C*_*α*_-atoms of the protein residues were selected for the RMSD calculations. The RMSD of the ligands was also calculated separately by selecting all ligand heavy atoms. The molecular mechanics Poisson-Boltzmann surface area (MMPBSA) approach was used to calculate the ligand binding energies^31^. In our MMPBSA calculations, we did not consider the membrane environment. However, the importance of taking it into account cannot be completely ignored. In this context, the limitations of our MMPBSA protocol are discussed in the “Discussion” section. The MMPBSA calculations were performed using the g_mmpbsa package developed under the Open-Source Drug Discovery Consortium (OSDD)^31^. It is a GROMACS tool for calculating the binding energy using the MMPBSA method without the entropic term. The ligand binding energy (*E*_*bind*_^total^) was estimated as the summation of the vdW interaction energy (*E*_*int*_^vdW^), electrostatic interaction energy (*E*_*int*_^elec^), polar solvation energy (*E*^polar^), and nonpolar solvation energy (*E*^non−polar^). The net nonbonded interaction energy (*E*_*int*_^int^), which is the summation of *E*_*int*_^vdW^ and *E*_*int*_^elec^, was calculated in vacuum. *E*^non−polar^ was estimated by using the nonpolar solvation model of the solvent accessible surface area (SASA). The last 10 ns of the 100 ns production run simulations were used to perform the binding energy and energy decomposition analysis. The dielectric constant was set to 2 for the calculation of the potential energy in vacuum. All other parameters were set to their default values. Furthermore, per-residue decomposition analyses were performed to obtain the energetic contributions from the amino acid residues to the total ligand binding energies. Additionally, the per-residue energetic contributions (*E*_*bind*_^res^) were decomposed into the molecular mechanics net nonbonded interaction energies (*E*^MM^), polar solvation energies (*E*^polar^) and nonpolar solvation energies (*E*^non−polar^).

## Supplementary Information

Supplementary Information.

## Data Availability

Raw data is available upon request to the corresponding author.
