# Supplementary material for: Bell-Evans model and steered molecular dynamics in uncovering the dissociation kinetics of ligands targeting G-protein-coupled receptors
Source: Sci Rep. 2022 Sep 24;12:15972. doi: 10.1038/s41598-022-20065-2 (PMC9509322; doi:10.1038/s41598-022-20065-2)
Supplement: Supplementary file 1 — Supplementary Information. [file 41598_2022_20065_MOESM1_ESM.pdf]

SUPPLEMENTARY INFORMATION

Bell-Evans Model and Steered Molecular Dynamics in Uncovering the Dissociation Kinetics of Ligands Targeting G-protein-coupled Receptors

Muhammad Jan Akhunzada[1], **Hyun Jung Yoon**[1,2], **Indrajit Deb**[1], **Abdennour Braka**[1], **Sangwook Wu** [1,2,*]

[1]R&D Center, PharmCADD Co. Ltd., 12F, 331, Jungang-daero, Dong-gu, Busan, 48792, Republic of Korea

[2] Department of Physics, Pukyong National University, Busan 48513, Republic of Korea

[*]s.wu@pharmcadd.com

PDB id	ligand	protein RMSD (nm)	ligand RMSD	R_G (nm)
3EML	ZMA241385	0.21 ± 0.02	0.11 ± 0.01	2.17 ± 0.01
2YDV	NECA	0.21 ± 0.04	0.06 ± 0.01	2.21 ± 0.01

Table S1. Average root mean square deviation (RMSD) and radious of gyration (R_G).

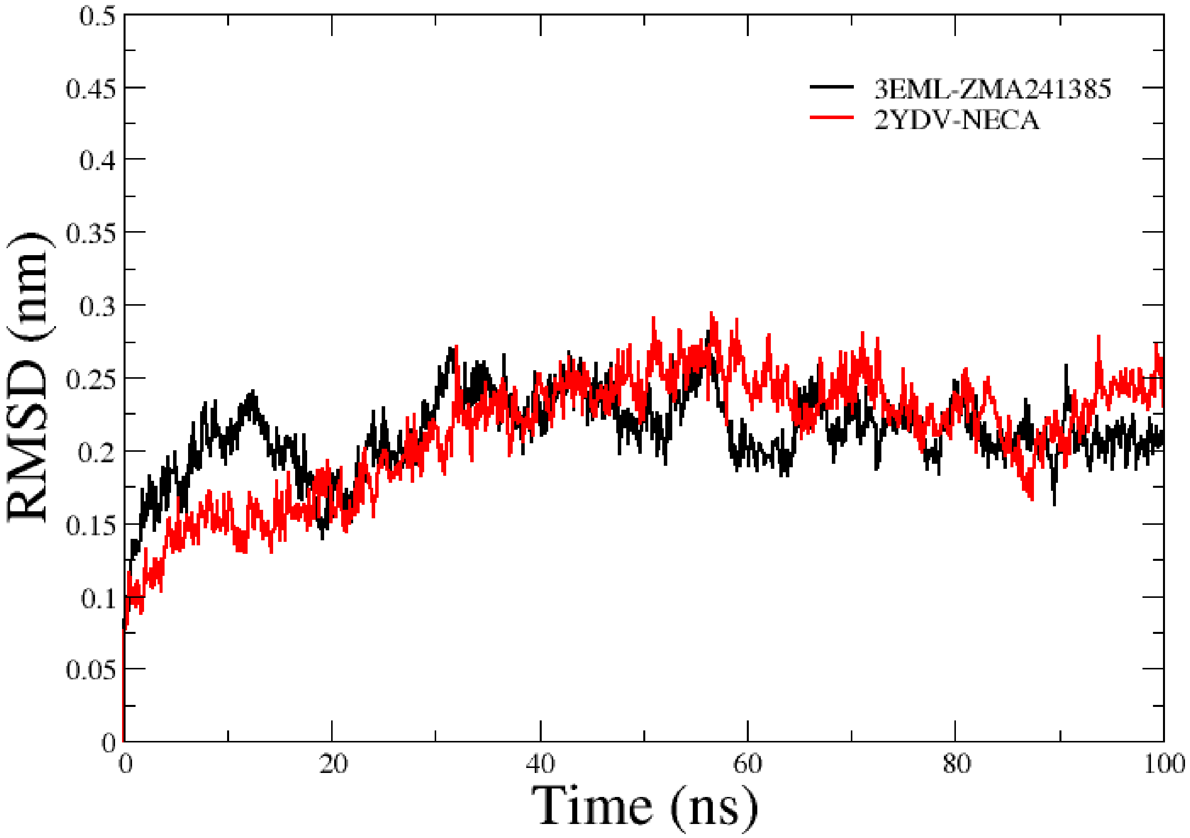

Figure S1. Time evolution of the root mean square deviations (RMSD) with respect to the initial coordinates of the C_α-atoms of the protein residues. 100 ns conventional MD simulations of the A2A ARs in complex with the ligands ZMA241385 (black) and NECA (red) were used for the RMSD calculations.

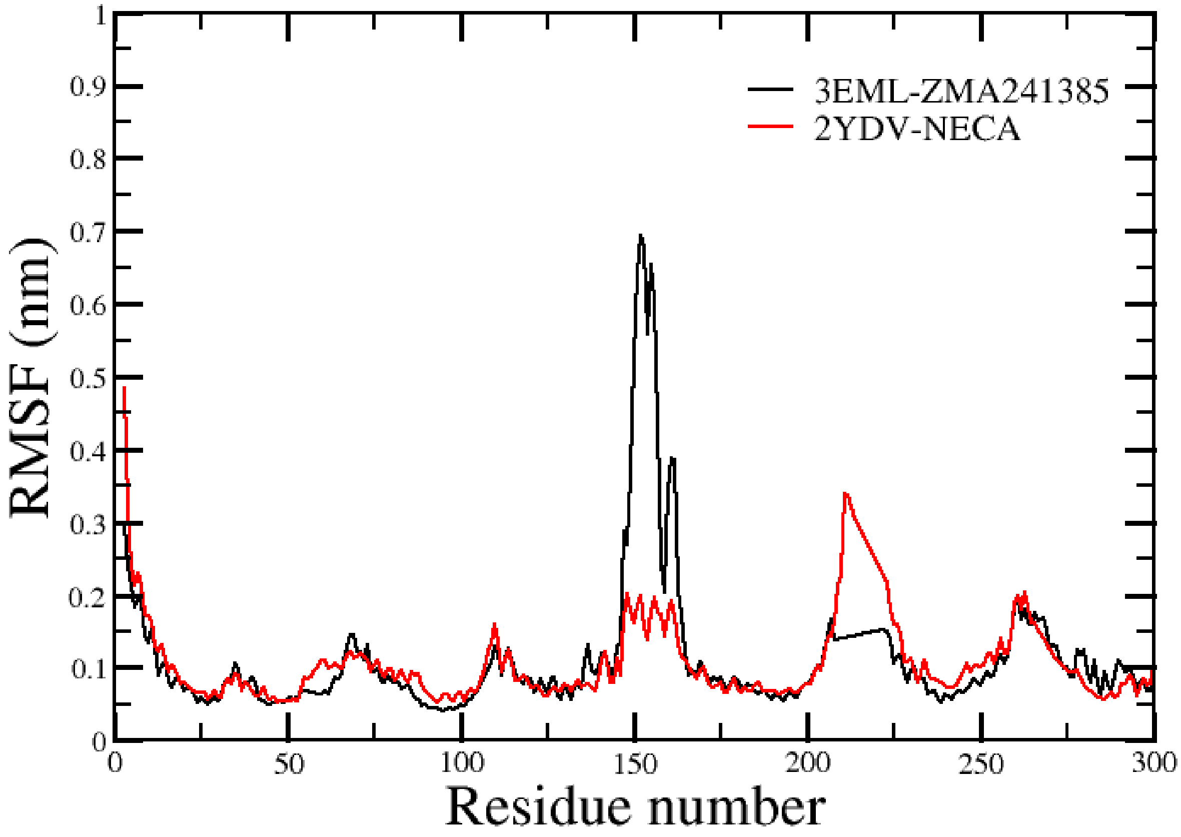

Figure S2. Residue-wise root mean square fluctuations (RMSF) considering the C_α-atoms of the protein residues. 100 ns conventional MD simulations of the A2A ARs in complex with the ligands ZMA241385 (black) and NECA (red) were used for the RMSF calculations.

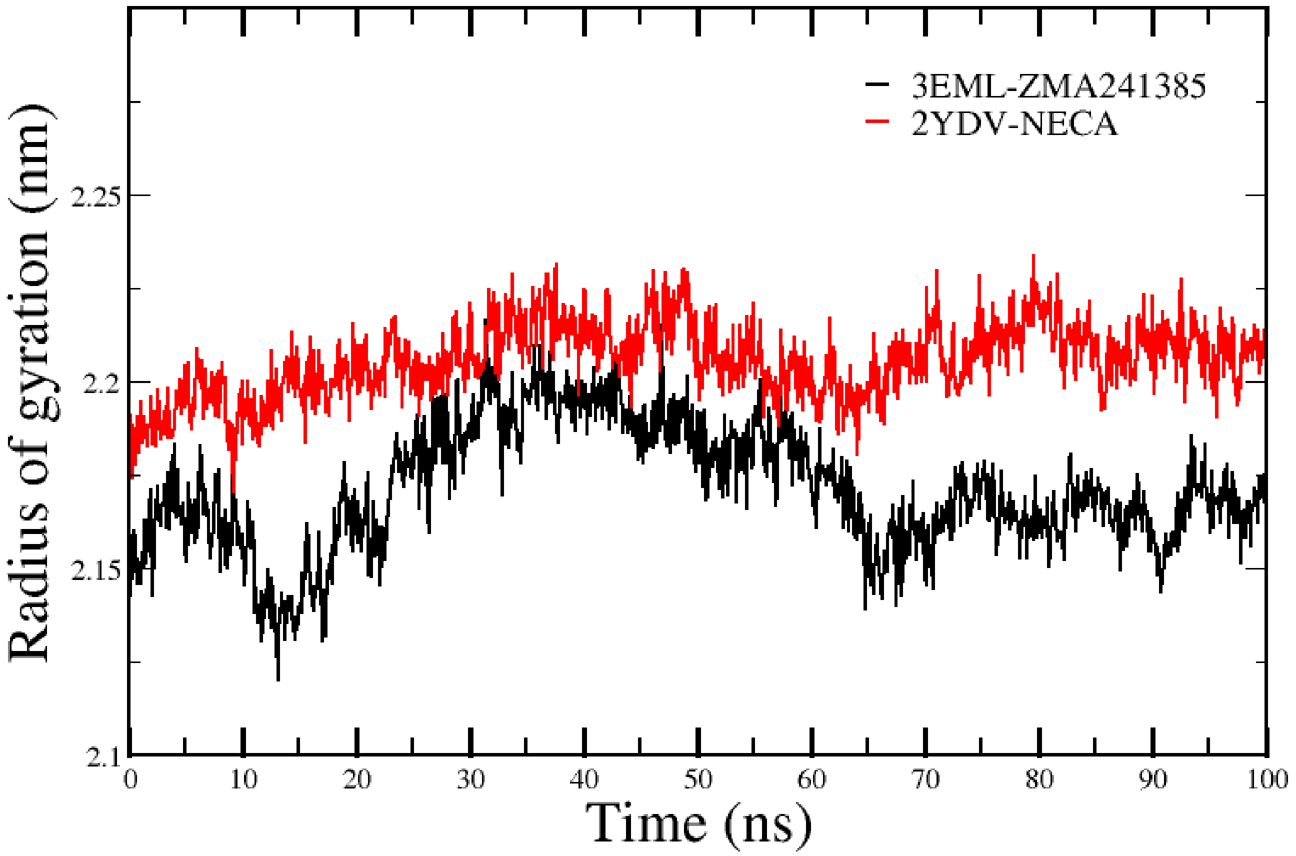

Figure S3. Time evolution of the radius of gyrations (R_G). 100 ns conventional MD simulations of the A2A ARs in complex with the ligands ZMA241385 (black) and NECA (red) were used for the R_G calculations.

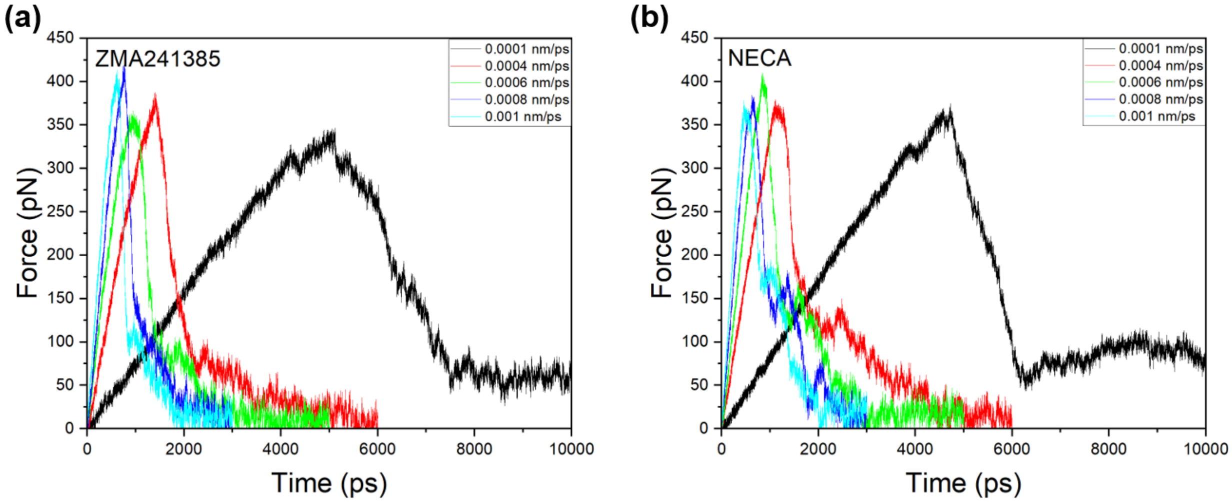

Figure S4. Average unbinding force profile at each of the pulling velocities for a) ZMA241385 and b) NECA ligand-receptor complexes. Unbinding forces were averaged over 41 replica simulations at 0.1 ps timestep along the SMD simulation trajectories.

PDB id	ligand	v (nm/ps)	$F_R^{\max}$ (pN)	$\mathrm{T}^{\max}$ (ps)
3EML	ZMA241385	0.0001	346.35	5120
		0.0004	387.32	1410
		0.0006	365.93	946
		0.0008	419.13	765
		0.0010	413.37	618
2YDV	NECA	0.0001	374.63	4733
		0.0004	379.23	1113
		0.0006	409.75	850
		0.0008	384.18	622
		0.0010	378.01	458

Table S2. Maximum unbinding force ($F_R^{\max}$) and the corresponding maximum time ($\mathrm{T}^{\max}$) estimated from the average force profile at each of the pulling velocities (v) for ZMA241385 and NECA ligand-receptor complexes. Unbinding forces were averaged over 41 replica simulations at 0.1 ps timestep along the SMD simulation trajectories.

residue	3EML-ZMA241385				2YDV-NECA			
	E_{bind}^{res} (kJ/mol)	E^{MM} (kJ/mol)	E^{polar} (kJ/mol)	$E^{\mathrm{non-polar}}$ (kJ/mol)	E_{bind}^{res} (kJ/mol)	E^{MM} (kJ/mol)	E^{polar} (kJ/mol)	$E^{\mathrm{non-polar}}$ (kJ/mol)
VAL84	-1.79 ± 0.08	-2.67 ± 0.09	1.15 ± 0.04	-0.27 ± 0.01	-3.22 ± 0.10	-5.79 ± 0.12	2.98 ± 0.06)	-0.41 ± 0.02
LEU85	-1.65 ± 0.10	-2.17 ± 0.10)	0.75 ± 0.02	-0.23 ± 0.01	-3.45 ± 0.10	-6.24 ± 0.14	3.08 ± 0.10	-0.29 ± 0.01
PHE168	-8.25 ± 0.15	-11.72 ± 0.16	4.54 ± 0.12	-1.07 ± 0.02	-8.15 ± 0.19	-12.26 ± 0.19	5.06 ± 0.14	-0.95 ± 0.02
GLU169	5.54 ± 0.78	-11.47 ± 0.51	17.62 ± 1.15	-0.61 ± 0.03	3.66 ± 0.38	-4.16 ± 0.27	8.22 ± 0.56	-0.40 ± 0.02
MET177	-2.98 ± 0.08	-3.74 ± 0.08	0.91 ± 0.05	-0.15 ± 0.01	-2.32 ± 0.07	-3.16 ± 0.09	1.21 ± 0.05	-0.37 ± 0.02
ASN181	0.99 ± 0.13	-1.33 ± 0.07	2.39 ± 0.13	-0.07 ± 0.01	4.58 ± 0.15	-2.53 ± 0.08	7.54 ± 0.14	-0.43 ± 0.01
TRP246	-2.77 ± 0.11	-3.14 ± 0.11	0.78 ± 0.05	-0.41 ± 0.01	-3.11 ± 0.11	-3.72 ± 0.14	1.32 ± 0.08	-0.71 ± 0.03
LEU249	-7.70 ± 0.11	-8.81 ± 0.11	1.72 ± 0.07	-0.61 ± 0.02	-0.53 ± 0.03	-0.50 ± 0.03	0.01 ± 0.02	-0.04 ± 0.01
HIS250	1.64 ± 0.13	-2.34 ± 0.10	4.24 ± 0.14	-0.26 ± 0.01	1.47 ± 0.21	-0.23 ± 0.06	1.83 ± 0.22	-0.13 ± 0.01
ASN253	3.88 ± 0.19	-11.45 ± 0.13	15.74 ± 0.15	-0.41 ± 0.01	0.07 ± 0.04	-0.80 ± 0.06	0.95 ± 0.08	-0.08 ± 0.01
MET270	-6.26 ± 0.18	-6.91 ± 0.21	1.50 ± 0.08	-0.85 ± 0.03	-0.32 ± 0.01	-0.17 ± 0.01	-0.15 ± 0.01	0.00 ± 0.00
ILE274	-4.19 ± 0.11	-4.59 ± 0.12	1.09 ± 0.03	-0.69 ± 0.02	-0.61 ± 0.02	-0.65 ± 0.03	0.05 ± 0.02	-0.01 ± 0.00

Table S3. Common residues within 5 Å of the ligands ZMA241385 and NECA in the binding pocket of the crystal structure of the A2A AR-ligand complex (PDB ids: 3EML and 2YDV, respectively) and their contribution (E_{bind}^{res}) to the total ligand binding energies ($E_{bind}^{\mathrm{total}}$). Decomposition of the E_{bind}^{res} into molecular mechanics net non-bonded interaction energies (E^{MM}), polar solvation energies (E^{polar}) and, non-polar solvation energies ($E^{\mathrm{non-polar}}$) are summarized.

PDB id	ligand	no. of atoms	lipids in upper layer	lipids in lower layer	no. of waters	box dimension	no. of K^+	no. of Cl^-
3EML	ZMA241385	111798	103	96	26751	90*90*147	73	83
2YDV	NECA	95659	104	99	35238	90*90*126	56	65

Table S4. Initial system parameters for the molecular dynamics (MD) simulations of the A2A ARs bound to the ligands ZMA241385 and NECA.

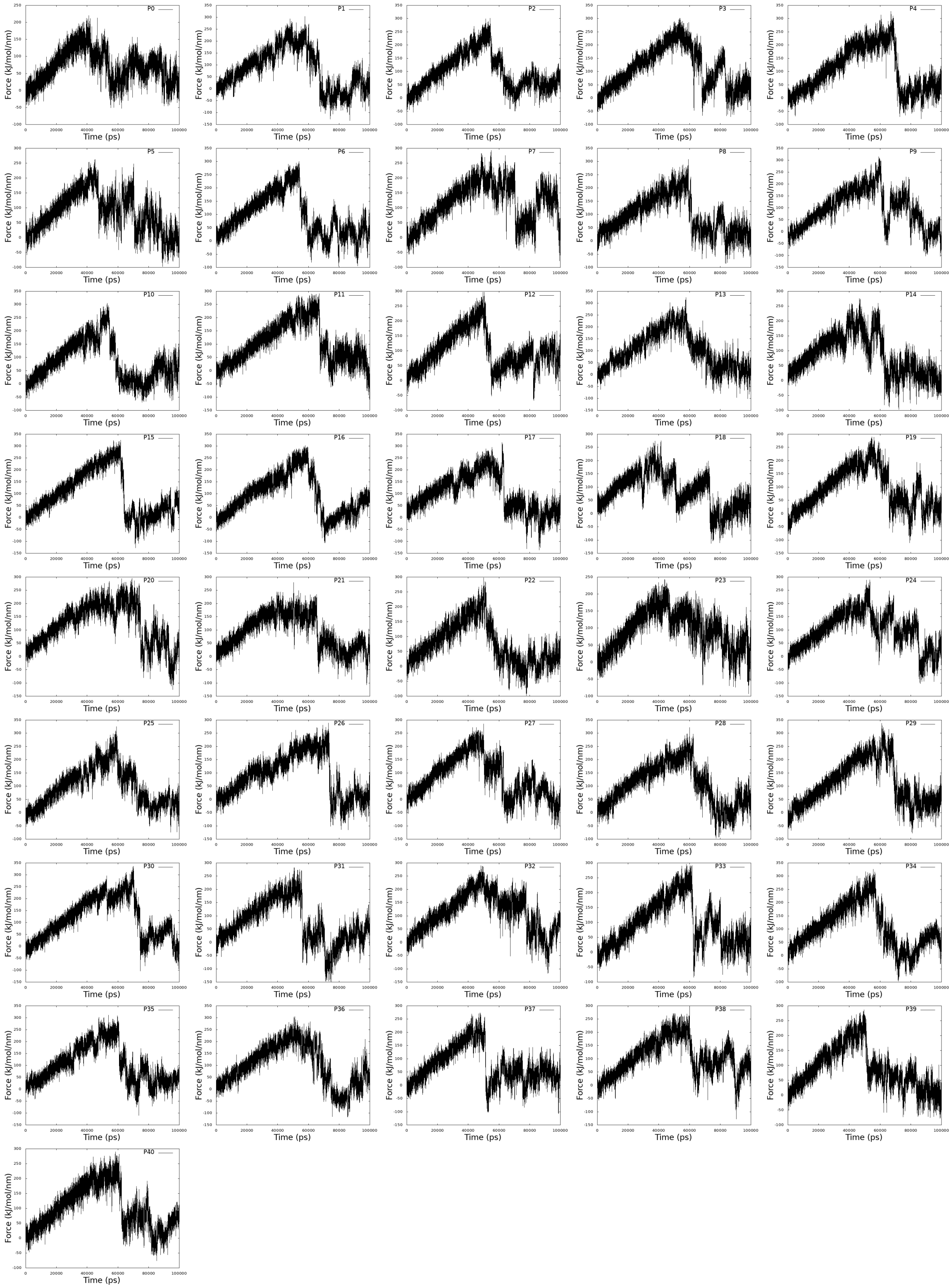

Figure S5. Unbinding force profiles for all the 41 replica SMD simulations of ZMA241385 ligand-receptor complexes at a pulling velocity of 0.0001 nm/ps.

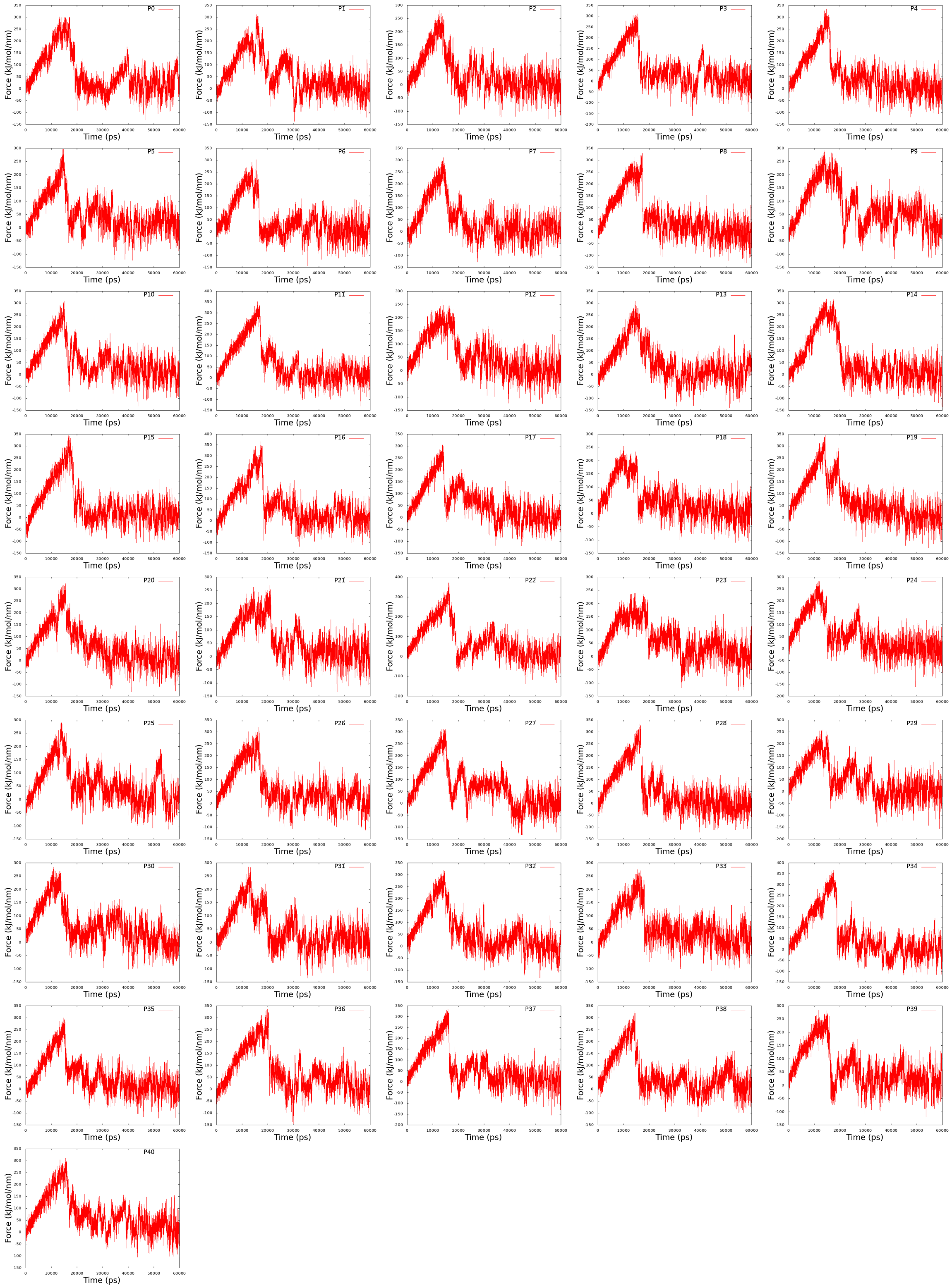

Figure S6. Unbinding force profiles for all the 41 replica SMD simulations of ZMA241385 ligand-receptor complexes at a pulling velocity of 0.0004 nm/ps.

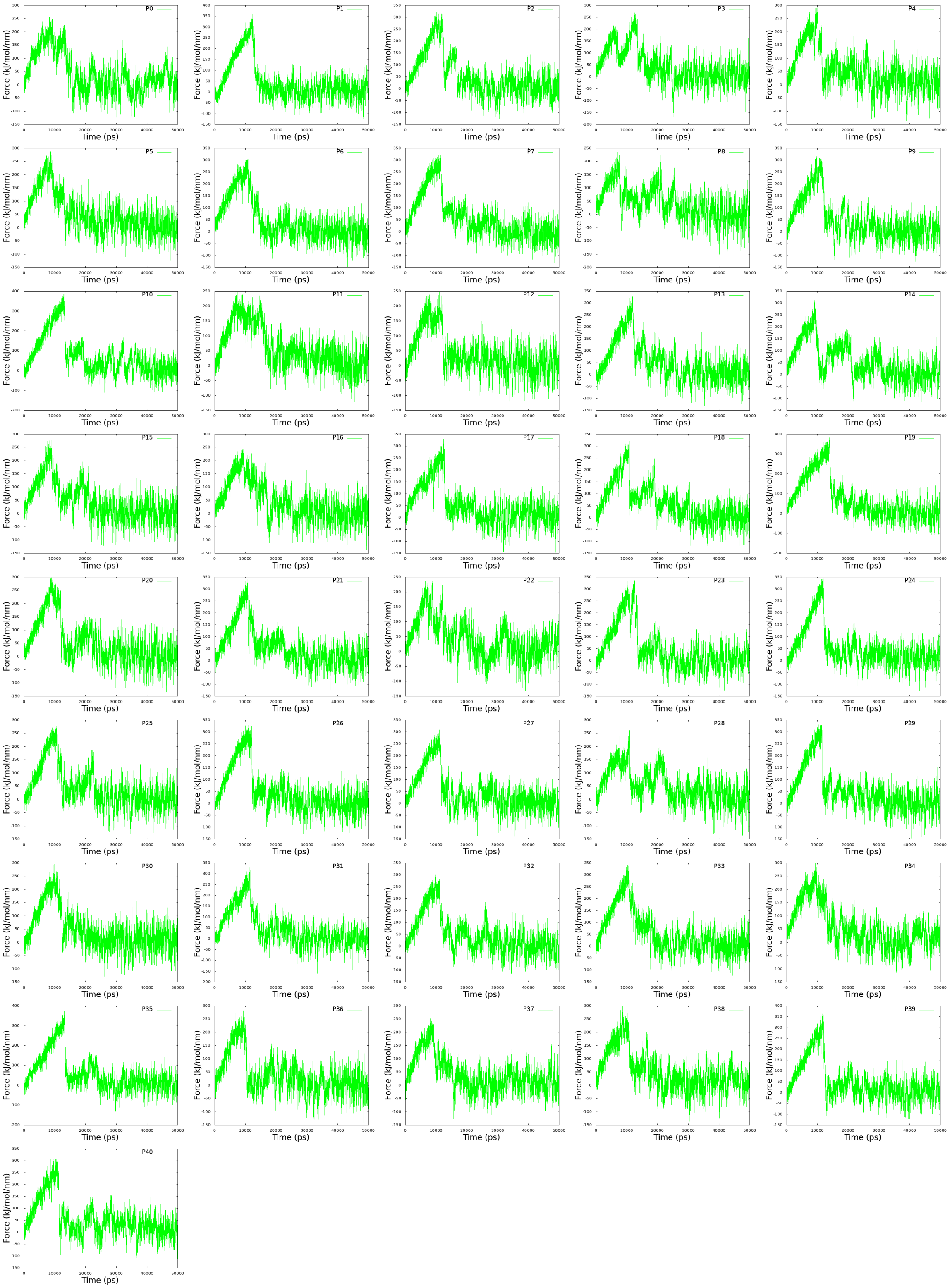

Figure S7. Unbinding force profiles for all the 41 replica SMD simulations of ZMA241385 ligand-receptor complexes at a pulling velocity of 0.0006 nm/ps.

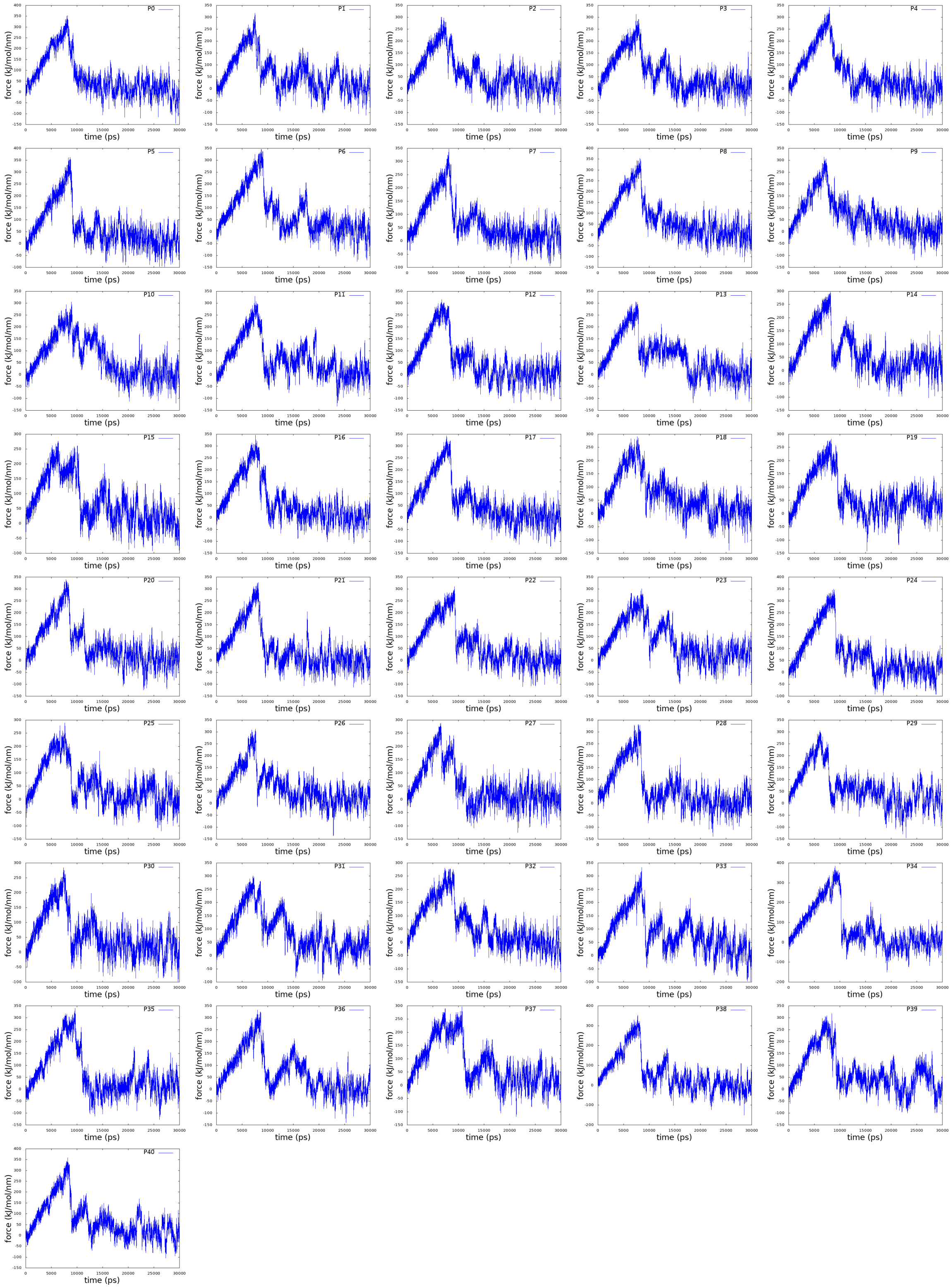

Figure S8. Unbinding force profiles for all the 41 replica SMD simulations of ZMA241385 ligand-receptor complexes at a pulling velocity of 0.0008 nm/ps.

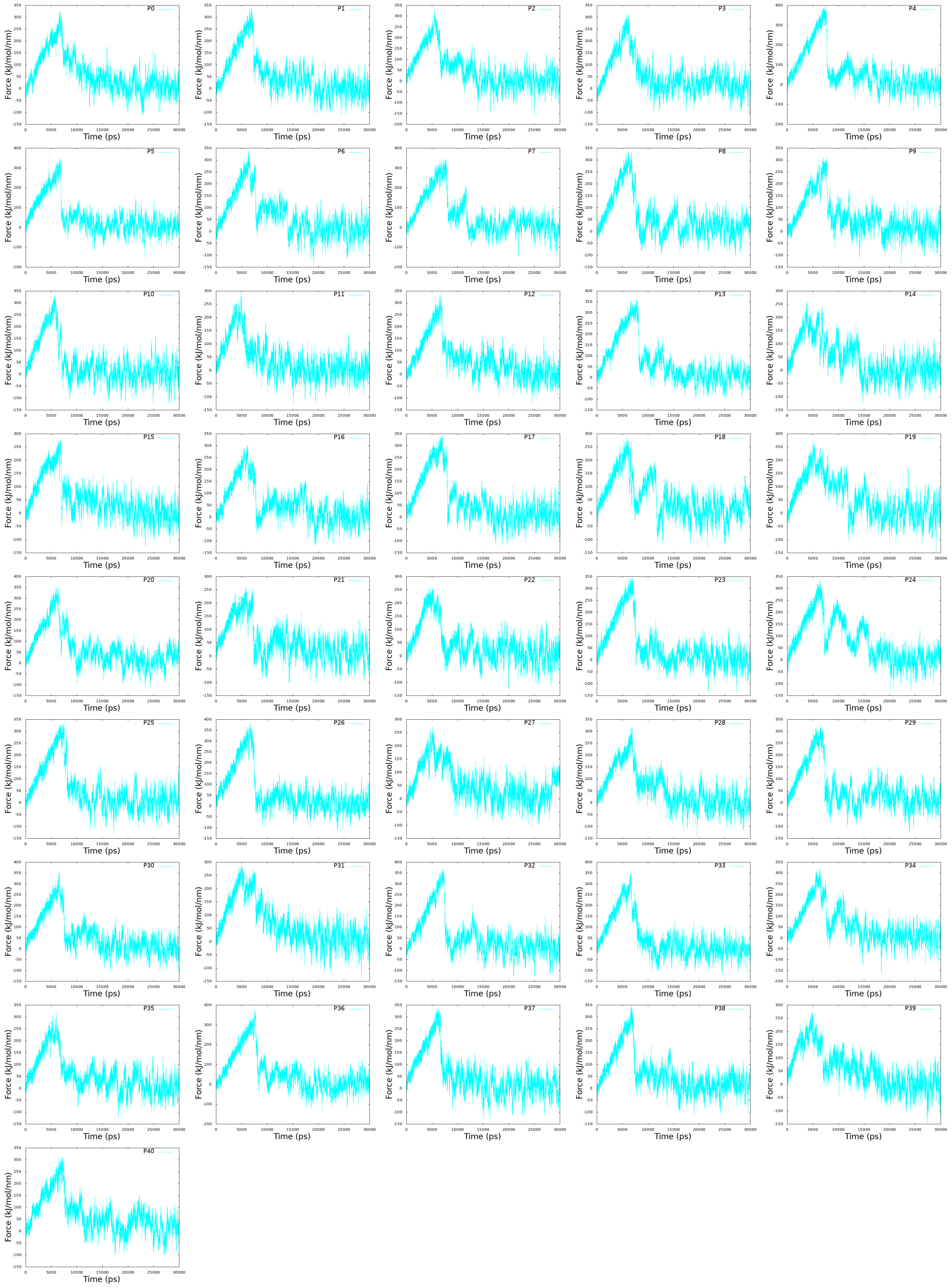

Figure S9. Unbinding force profiles for all the 41 replica SMD simulations of ZMA241385 ligand-receptor complexes at a pulling velocity of 0.0010 nm/ps.

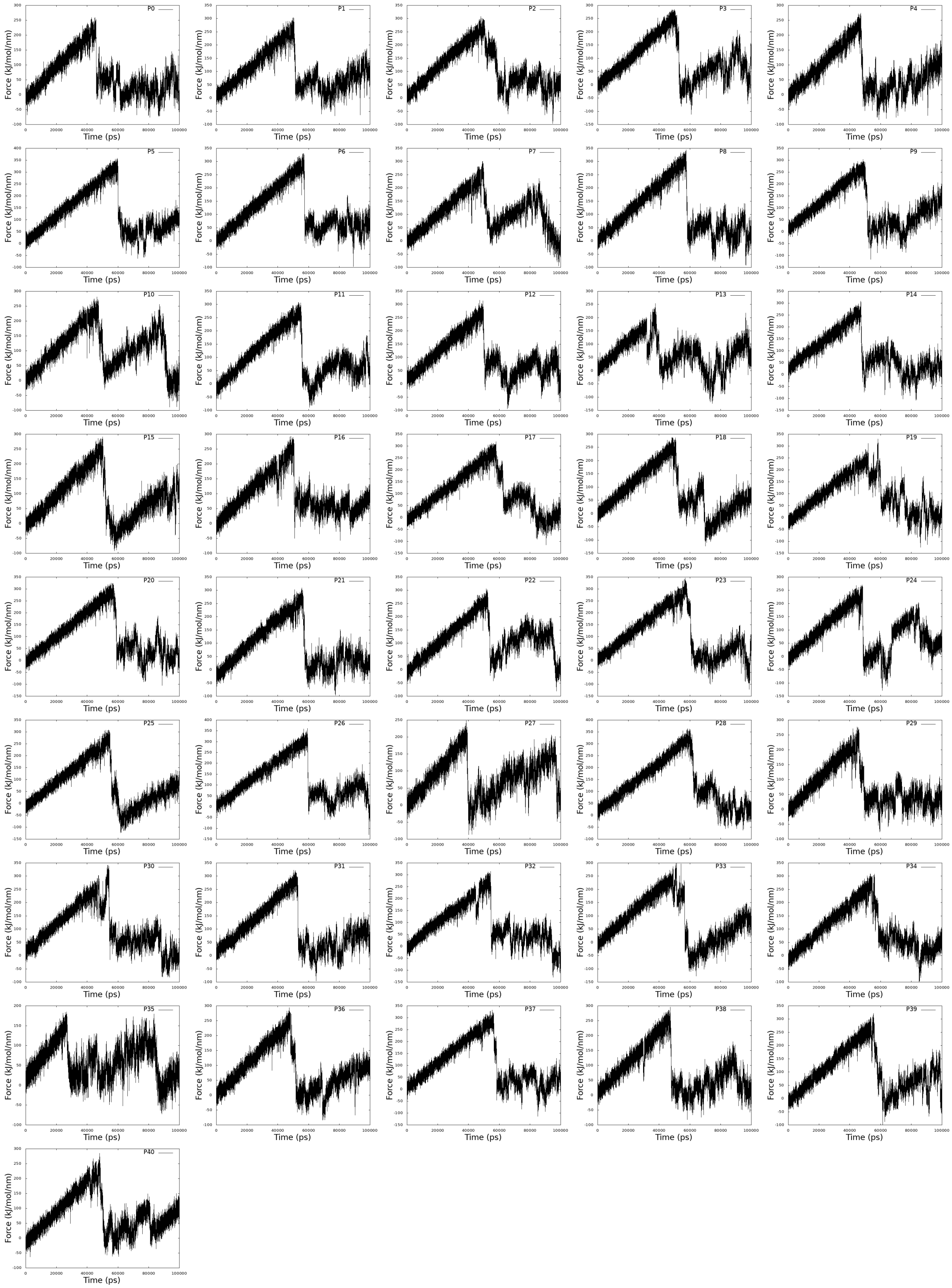

Figure S10. Unbinding force profiles for all the 41 replica SMD simulations of NECA ligand-receptor complexes at a pulling velocity of 0.0001 nm/ps.

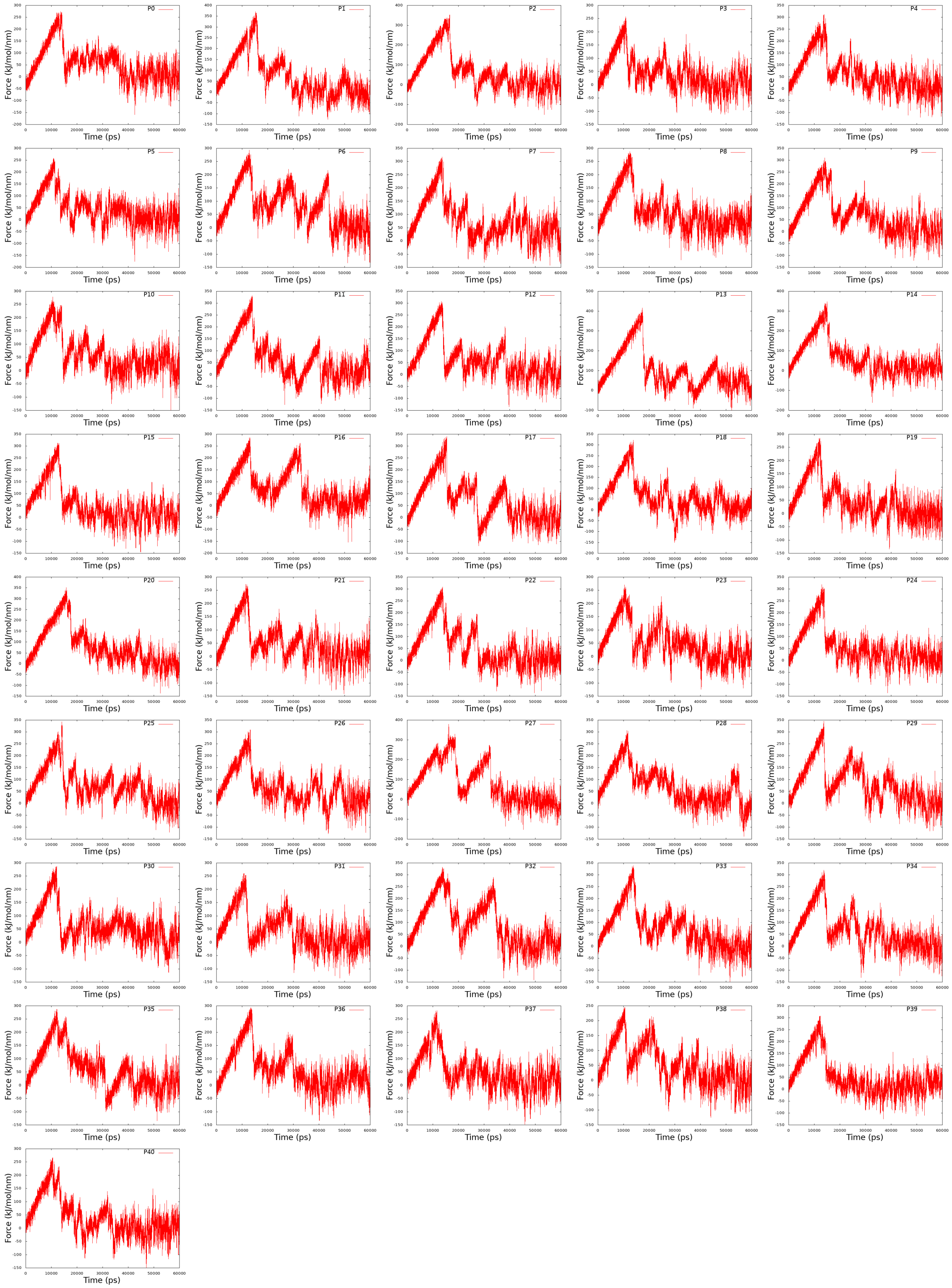

Figure S11. Unbinding force profiles for all the 41 replica SMD simulations of NECA ligand-receptor complexes at a pulling velocity of 0.0004 nm/ps.

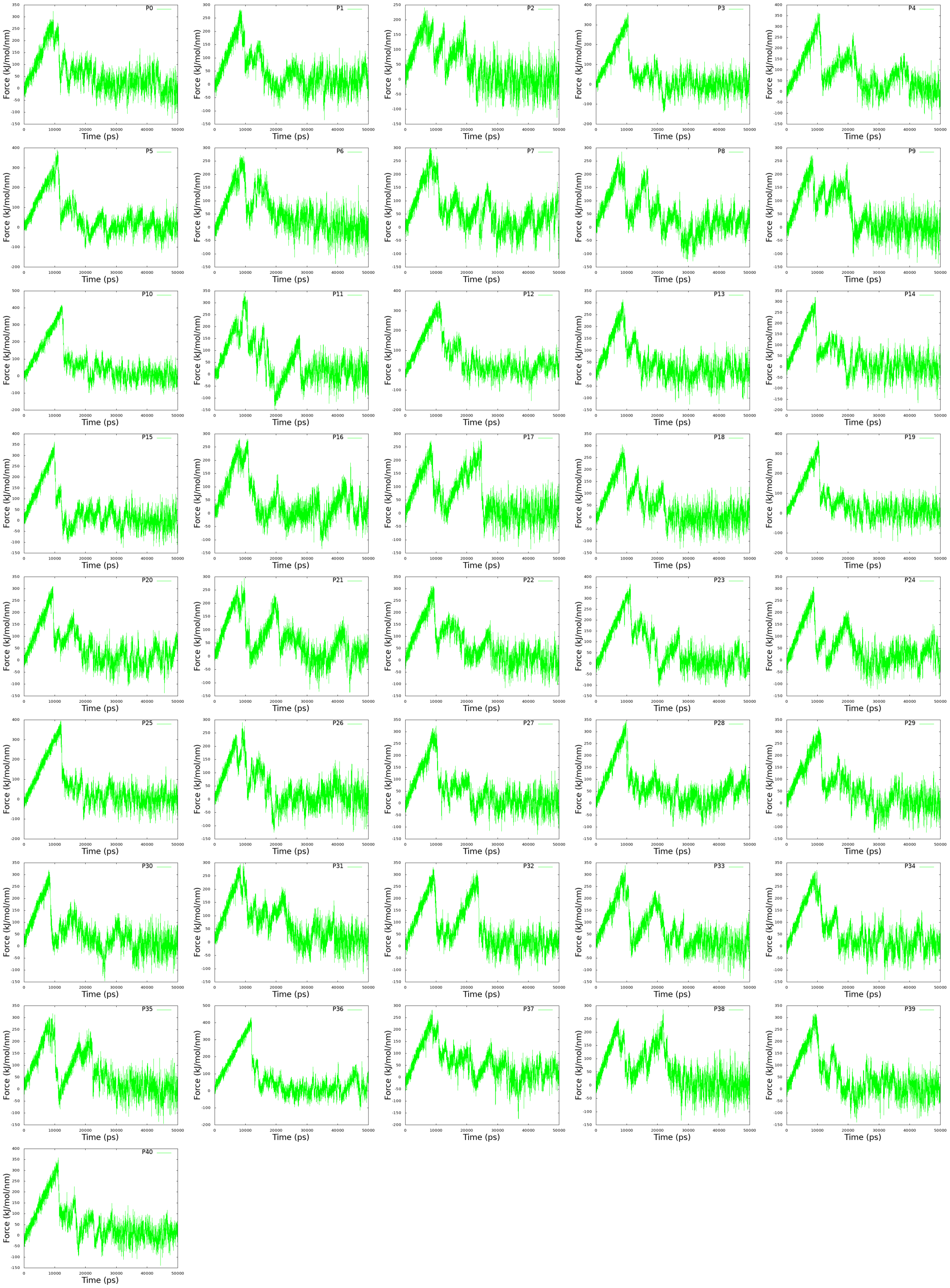

Figure S12. Unbinding force profiles for all the 41 replica SMD simulations of NECA ligand-receptor complexes at a pulling velocity of 0.0006 nm/ps.

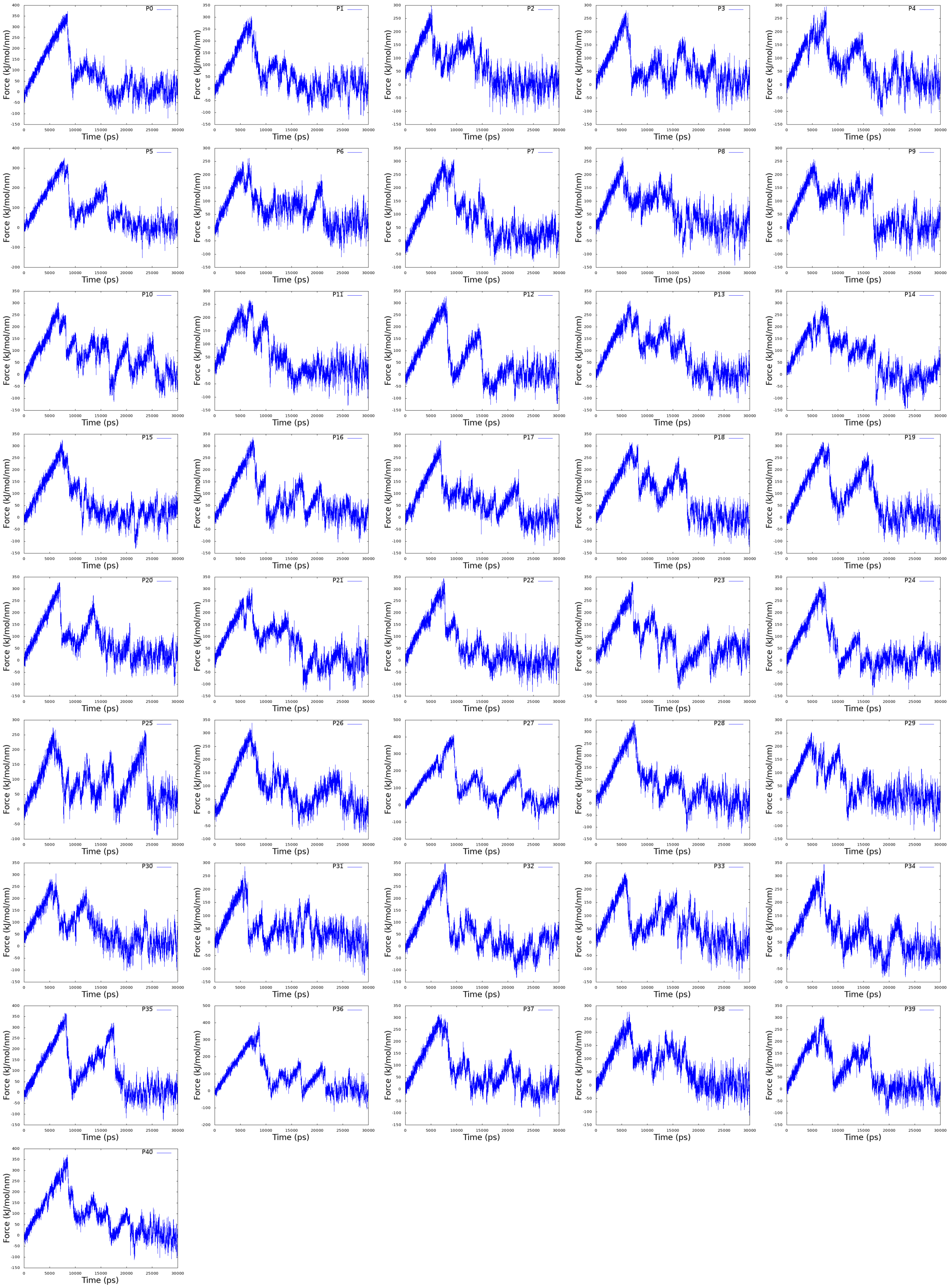

Figure S13. Unbinding force profiles for all the 41 replica SMD simulations of NECA ligand-receptor complexes at a pulling velocity of 0.0008 nm/ps.

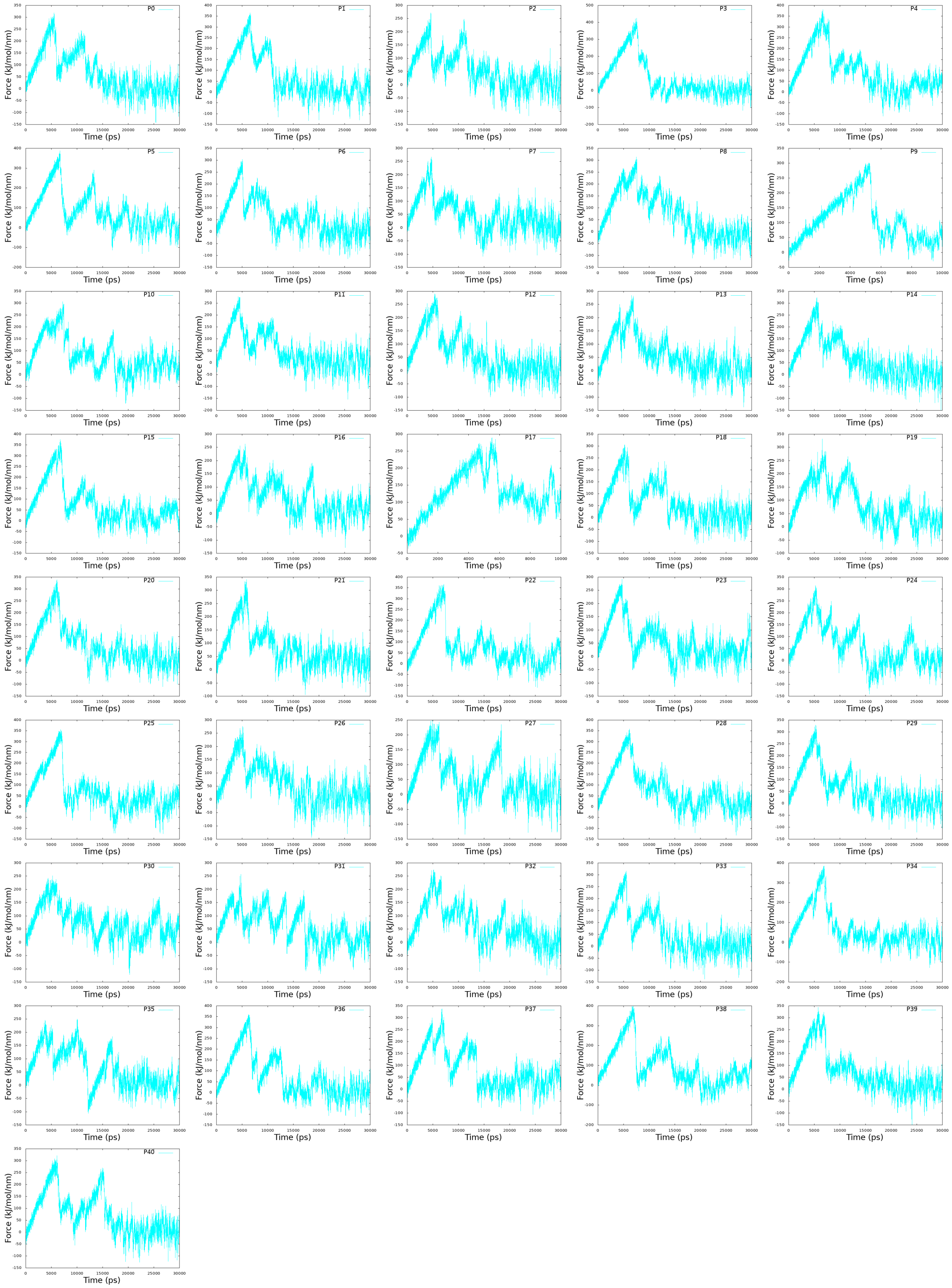

Figure S14. Unbinding force profiles for all the 41 replica SMD simulations of NECA ligand-receptor complexes at a pulling velocity of 0.0010 nm/ps.

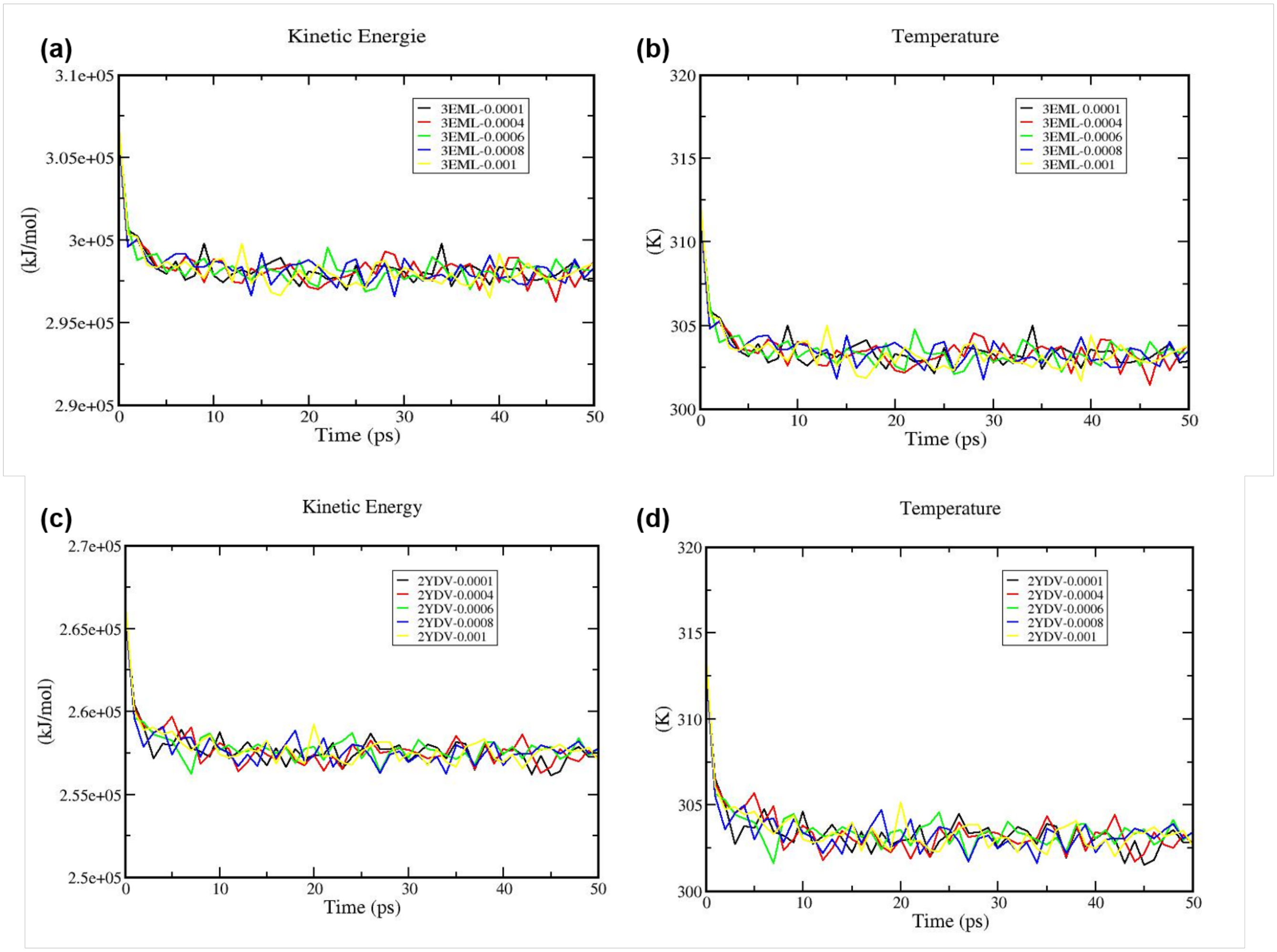

Figure S15. Time evolutions of kinetic energy and temperature for the 50 ps equilibration runs of the A2A ARs in complex with the ligands ZMA241385 (a and b, top panel) and NECA (c and d, bottom panel). Time evolutions are shown for one representative replica out of 41 replica simulations at different pulling velocities.
